# Improving stereoselectivity of phosphotriesterase (PTE) for kinetic resolution of chiral phosphates

**DOI:** 10.3389/fbioe.2024.1446566

**Published:** 2024-07-30

**Authors:** Nongluck Jaito, Suthathip Phetlum, Titiporn Saeoung, Thanat Tiyasakulchai, Nitipol Srimongkolpithak, Tanaporn Uengwetwanit

**Affiliations:** National Center for Genetic Engineering and Biotechnology (BIOTEC), National Science and Technology Development Agency (NSTDA), Khlong Luang, Thailand

**Keywords:** phosphotriesterase, PTE, biocatalyst, active pharmaceutical ingredient, API, specificity

## Abstract

Specific stereoisomer is paramount as it is vital for optimizing drug efficacy and safety. The quest for the isolation of desired stereoisomer of active pharmaceutical ingredients or key intermediates drives innovation in drug synthetic and biocatalytic methods. Chiral phosphoramidate is an important building block for the synthesis of antiviral drugs such as remdesivir and sofosbuvir. Given the clinical potency of the (*S*p)-diastereomer of the drugs, an enzyme capable of completely hydrolyzing the (*R*p)-diastereomer is needed to achieve the purified diastereomers *via* biocatalytic reaction. In this study, protein engineering of phosphotriesterase (PTE) was aimed to improve the specificity. Employing rational design and site-directed mutagenesis, we generated a small library comprising 24 variants for activity screening. Notably, W131M and I106A/W131M variants demonstrated successful preparation of pure (*S*p)-diastereomer of remdesivir and sofosbuvir precursors within a remarkably short hydrolysis time (<20 min). Our work unveils a promising methodology for producing pure stereoisomeric compounds, utilizing novel biocatalysts to enable the chemoenzymatic synthesis of phosphoramidate nucleoside prodrugs.

## Introduction

Biocatalysis has emerged as a highly valuable tool, revolutionizing the production of intermediates, desired pure diastereomer building blocks, and active pharmaceutical ingredients (APIs) in the pharmaceutical industry (Alcántara et al., 2022; Rossino et al., 2022). Enzymes, as biocatalysts, offer several advantages over traditional chemical synthesis. Due to their specificity, efficiency, and environmental friendliness, enzymes promote greener and more efficient manufacturing processes (Adams et al., 2019; Sheldon et al., 2020; Lewis et al., 2023). Moreover, the U.S. Food and Drug Administration enforces strict rules on chiral drugs due to the significant impact of chirality on pharmaceutical properties (FDA, 1992). Different enantiomers can have varying efficacy and safety profiles, making it crucial to ensure the purity and proper identification of the active form. The regulatory environment compels manufacturers to promote enantiopure drugs whenever feasible. The conventional methods used for chiral separation, such as crystallization and chiral chromatography, are challenging and inefficient in terms of both speed and scalability (Rougeot and Hein, 2015; Sui et al., 2023). This further advocates for utilization of enzymes as enantioselective catalysts.

**FIGURE 1:**
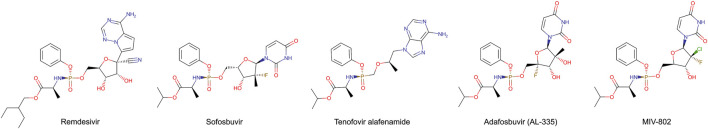
FDA-approved and clinical candidate phosphoramidate nucleotide prodrugs.

Stereoisomerically pure phosphoramidate nucleoside prodrugs (ProTide) represent an advancement in synthetic intermediates for nucleoside analogues (Figure 1) used to treat viral infection and cancer (Dousson, 2018; Slusarczyk et al., 2018; Sabat et al., 2022). Designing ProTide compounds is to enhance the pharmacological properties of nucleoside analogs, such as improved bioavailability, cell permeability, and resistance to enzymatic degradation within cells (Mehellou et al., 2018). The FDA approved ProTide are such as remdesivir, and sofosbuvir. Many ProTide have a stereogenic phosphorus center thus the therapeutic effect of the ProTide is predominantly attributed to one stereoisomer. For examples, the clinical prodrug form of remdesivir predominantly consists of the (*S*p)-diastereomer because of its higher selectivity and broader therapeutic range (Liang et al., 2020). Similarly, (*S*p)-diastereomer of sofosbuvir is more potent than the (*R*p)-diastereomer. (*S*p)-diastereomer of sofosbuvir demonstrates activity 18 times higher than the (*R*p)-diastereomer against hepatitis C (Sofia et al., 2010). As such, efficient methods for the chemical synthesis and isolation of stereochemically pure ProTides are imperative.

**FIGURE 2:**
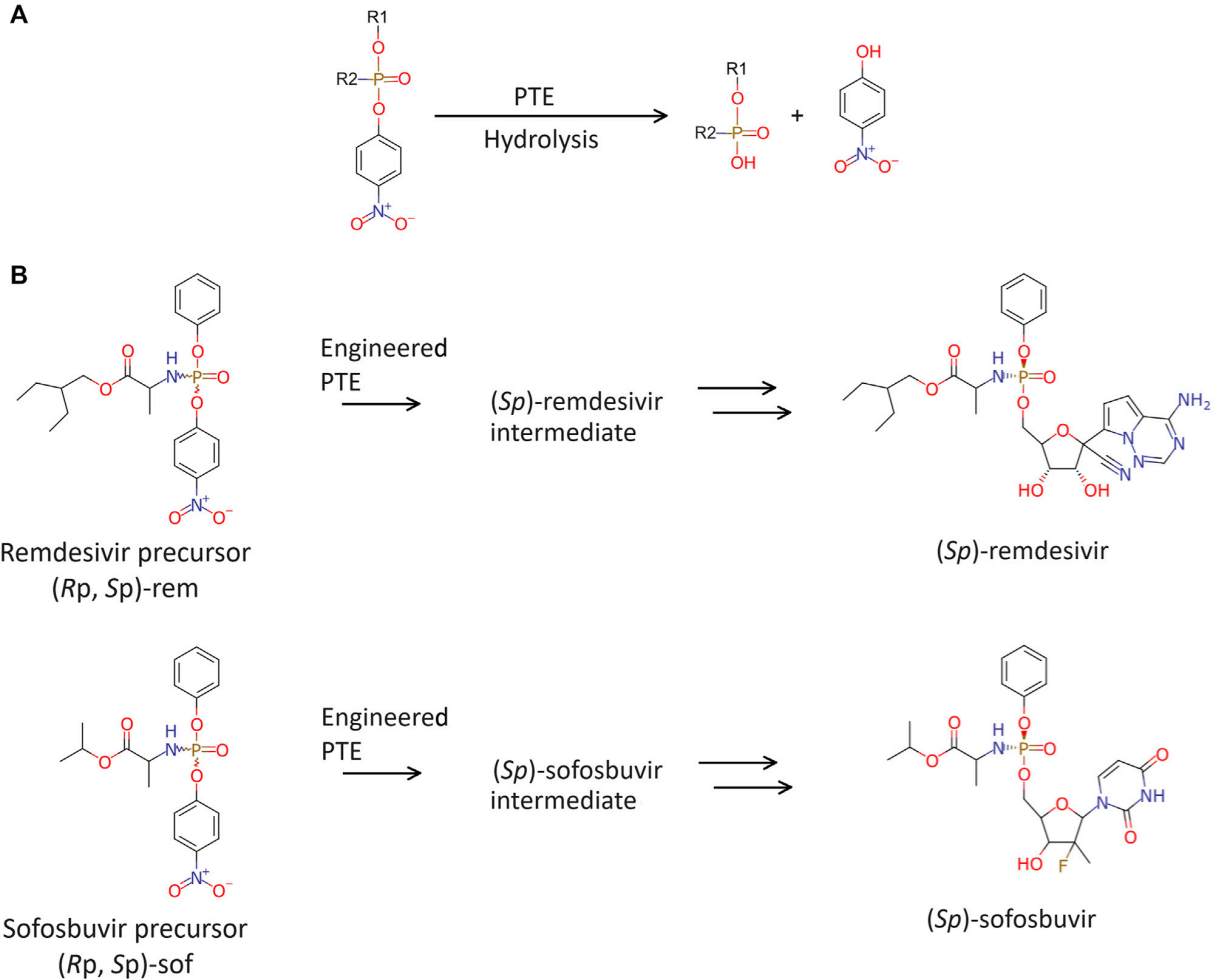
Enzymatic hydrolysis. **(A)** Phosphotriesterase (PTE) catalyzes hydrolysis of phosphate agents. **(B)** Brief chemoenzymatic synthetic route for ProTides. The preferred PTE should exhibit *Rp*-selective hydrolysis of remdesivir and sofosbuvir precursor.

Recently, engineered phosphotriesterase (PTE also known as parathion hydrolase) from *Brevundimonas diminuta* (formerly known as *Pseudomonas diminuta*) was reported toward purification of precursors for ProTide (Xiang et al., 2019; Bigley et al., 2020). PTE catalyzes the hydrolysis of *p-*nitrophenyl phosphotriesters with substituents at the phosphorus center (Figure 2A). Variants of PTE were screened to specifically hydrolyze either (*R*p)- or (*S*p)-diastereomeric precursor of remdesivir and sofosbuvir, which are hereinafter referred to as (*R*p, *S*p)-rem and (*R*p, *S*p)-sof, respectively. Variant G60A showed 165-fold preference for hydrolyzing the (*R*p)-sof. While a variant In1W (F132L/H254S/H257W/257-258insSAIGLDPIPN) impressively exhibited 1,400-fold for (*S*p)-sof (Xiang et al., 2019). In1W also exhibited >200-fold preference for hydrolyzing (*S*p)-rem. On the other hand, G60A did not demonstrate any observable stereoselective hydrolysis of (*R*p)-rem (Bigley et al., 2020). Stereoselective PTE toward the counterpart of the desired stereoisomer could enhance the production of highly pure stereoisomer precursors for ProTide synthesis. In case of preparative stereoisomer isolation of remdesivir and sofosbuvir, specific hydrolysis of (*R*p)-diastereomer would provide the pure (*S*p)-diastereomer which is the potent isoform (Figure 2B). Although previous studies have demonstrated remarkably success in isolating purified diastereomers, challenges remain in improving hydrolysis of (*R*p)-diastereomer of these prodrugs.

In this study, a small numbers of PTE variants were rationally designed and prepared to screen stereoselectivity toward (*R*p)-diastereomer of remdesivir and sofosbuvir precursors ((*R*p)-rem and (*R*p)-sof). The mutants with the improved specific activity were subjected to biochemical characterization in detail. We successfully obtained PTE variants that showed improved enantioselectivity toward both (*R*p)-rem and (*R*p)-sof. These findings underscore the potential of the variants for producing other stereoisomerically pure ProTides and organic compounds.

## Materials and methods

### Structural analysis and molecular docking

Structural analysis and molecular docking were employed to investigate the interactions between PTE and substrates. The complex structure of PTE and paraoxon analog was retrieved from the protein databank (PDB ID: 1DPM) (Vanhooke et al., 1996; Berman et al., 2000). Protein and ligand molecules were prepared with default structural preparation and protonation procedure in MOE (Molecular Operating Environment) program (Chemical Computing Group ULC, 2023). Molecular docking was performed using Triangle Matcher and GBVI/WSA rescoring. Visualization of the docked complexes facilitated the identification of interactions between the protein and ligand molecules and key residues involved in binding. Molecular dynamic (MD) simulation was used to validate the binding interactions between the docked poses and proteins (Supplementary data).

### Plasmid construction and PTE variants preparation

The metalloenzyme PTE derived from *B. diminuta* demonstrates remarkable efficiency in hydrolyzing diverse compounds. However, the investigation of PTE has faced challenges due to the inefficient expression of its recombinant form, despite its significant potential. This study utilizes an evolved variant of PTE, known for its successful heterologous expression, as the standard reference, denoted as wild type (WT), to address this issue (Roodveldt and Tawfik, 2005). The nucleotide sequence was obtained from Genbank (accession number: KU746636.1) and synthesized by GenScript without the leader peptide-coding fragment (99 bp) at N-terminal and tagged with six consecutive histidine residues (6×His) at C-terminal. The target gene was inserted into pMAL.c5x vector at *Nde*I and *Hind*III size (pMAL.c5x-WT-PTE).

In1W-PTE (F132L, H254S, H257W, and insertion of SAIGLDPIPN between amino acid residues at 257 and 258) was synthesized by GenScript and transformed into *Escherichia coli* BL21DE3 cells. Other PTE variants were constructed using the overlap extension PCR. Recombinant plasmid pMAL.c5x-WT-PTE was used as a PCR template. To introduce a single-site mutation *via* substitution, two pairs of primers were designed: (i) a forward-*Nde*I and a reverse primer tailored for the desired mutation, and (ii) a reverse-*Hind*III primer and a forward primer tailored for the intended mutation (Supplementary Table S1). The obtained PCR products were utilized as the template for the second PCR procedure, employing the forward-*Nde*I and reverse-*Hind*III primer pair. The PCR reactions were performed in the following order: initial denaturation for 5 min at 95°C, followed by 30 cycles of 95°C for 30 s, 55°C for 30 s, 72°C for 1 min, and final extension 72°C for 7 min. Each PTE mutant was inserted into the pMAL.c5x vector at the *Nde*I and *Hind*III site and subsequently transformed into *E. coli* DH5α. Randomly selected colonies were sequenced to ensure the presence of amino acid substitutions. The resulting recombinant plasmids were then used to transform into *E. coli* BL21DE3 cells, allowing for protein expression upon isopropyl thio-ß-D-galactoside (IPTG) induction.

## Screening for diastereoselective hydrolytic PTE variants

The expression of host cells harboring pMAL.c5x plasmid encoding the mutants PTE were cultured in 50 mL of Luria broth (LB) containing ampicillin 50 μg/mL at 37°C and 200 rpm. The target protein expression was induced by 0.2 mM IPTG when the optical density at 600 nm (OD_600_) reached 1.0-1.3. After that, the culture was incubated at 16°C for 18–20 h. The bacteria cells were harvested by centrifugation at 8,000 rpm for 10 min and resuspended in 5 mL of 50 mM Tris-HCl buffer (pH 8.0). The crude enzyme was extracted by sonication and removed cell debris by centrifuging at 17,000 rpm for 15 min at 4°C. The supernatant was then screened for an enantioselective variant.

To screen the enantioselectivity for hydrolysis of sofosbuvir and remdesivir precursor, the samples were performed and investigated by colorimetric assay. The reaction mixture (total volume 1 mL) contained 200 µg of crude enzyme extract, 50 mM CHES (pH 9.0), 0.1 mM CoCl_2_, and 60 µM substrate (two diastereomer of two precursors including (*S*p)-sof, (*S*p)-rem, (*R*p)-sof or (*R*p)-rem dissolved in 100% DMSO). The reaction was incubated at room temperature (25°C) for 60 min, then detected *p*-nitrophenol at 400 nm. The substrate complete hydrolysis with KOH was defined as 100% hydrolysis and was the control of the experiment. Each analysis was conducted in triplicate.

### Purification of recombinant PTE

The expression system for studying the enzyme kinetics of PTE was changed from pMAL.c5x plasmid, which contained MBP, to pET28a. While the fusion of PTE with MBP can enhance protein solubility, the relatively large size of MBP (42 kDa) (Raran-Kurussi and Waugh, 2012) may interfere with kinetic activity of the PTE. PTE has a molecular weight of ∼36 kDa (Bigley and Raushel, 2019). To facilitate biochemical characterization of PTE, MBP cleavage was achieved using the protease Factor Xa (Thermo Fisher Scientific Inc., United States) over a period of several days. Given the inherent instability of PTE, mutant PTE genes were subsequently integrated into the pET28a plasmid at the identical restriction site positioned between *Nde*I and *Hind*III sites. The constructed plasmids were transformed into *E. coli* BL21DE3 cells for recombinant PTE expression. The host cells were cultured in 1 L of LB containing kanamycin 50 μg/mL at 37°C and 200 rpm. The cultured cells reached OD_600_ 0.4-0.6, then added IPTG 0.1 mM, decreased the temperature to 16°C, and cultured for 20 h. The cultured cells were harvested and sonicated. The clear supernatant (100 mL) was added imidazole to final concentration of 20 mM and filtered through 0.2 µm before being applied to a HiPrep FF 16/10 column (GE Healthcare) which was also equilibrated by the equilibration buffer (20 mM Tris-HCl, pH 8.0 contained 0.1 M NaCl and 20 mM imidazole). The column was washed with the same buffer at a flow rate of 5 mL/min and the bound protein was eluted with a stepwise gradient imidazole (20–500 mM). The protein pattern of the eluted protein fractions was analyzed by using 12% sodium dodecyl sulfate-polyacrylamide gel electrophoresis (SDS-PAGE). All purified PTE fractions were pooled and dialyzed against 50 mM Tris-HCl buffer (pH 8.0) at 4°C with gentle stirring for desalting. The dialyzed enzyme was concentrated by ultrafiltration and immediately kept the enzyme in 20% glycerol at 4°C.

### Determination of enzyme kinetic parameters

Preliminary assays with varying enzyme concentrations were conducted, and the initial rates were measured to determine the appropriate concentration of enzyme in kinetic studies (Supplementary Table S2). The optimal concentration is selected from the linear range of the initial rate *versus* enzyme concentration curve, ensuring reliable measurements without saturation. This process balances sensitivity and linearity for accurate kinetic analysis.

The purified PTEs were subjected to characterization of steady-state kinetic constants using *R*p- or *S*p-diastereomer of sofosbuvir precursor ((*R*p, *S*p)-sof) and remdesivir precursors ((*R*p, *S*p)-rem). The reaction mixtures (1 mL) consisted of 50 mM CHES (pH 9.0), 0.1 mM CoCl_2_, and substrate concentrations ranging from 10 μM to 250 µM (dissolved in 100% DMSO). Incubation was carried out at 30°C for 10 min. Reactions were initiated by adding the appropriate enzyme concentration (Supplementary Table S2) and monitoring the release of *p*-NP at 400 nm with a spectrophotometer. All experiments were conducted in triplicate, with a control reaction performed in the absence of an enzyme. The Michaelis-Menten constant (*k*_m_) and maximum velocity (*V*_max_) were determined from the Lineweaver-Burk plot using a computer program provided using GraFit Version 7 Software. Thus, the catalytic efficiency of the enzyme (*k*_cat_/*K*_m_) was calculated using the equation: *k*_cat_ = *V*_max_/[E_t_], where *k*_cat_ is the turnover number, [E_t_] is the total enzyme concentration in moles (with the molecular weight of PTE being 38,128.55 g/mol), and V_max_ is maximum velocity or the reaction rate when the enzyme is fully saturated by the substrate. To facilitate the comparison of substrate stereoselectivity, the ratio of the kinetic parameters was calculated as follows.

$$K_{m}\text{ ratio}=K_{m\;\left(Rp\;substrate\right)}\;/\;K_{m\;\left(Sp\;substrate\right)}$$

A *K*_m_ ratio less than one indicates that *R*p substrate exhibits higher affinity for the enzyme compared to *S*p substrate. Conversely, a ratio greater than one suggests that *S*p substrate has higher affinity. A ration close to one indicates similar affinities for both isoforms.

$$k_{cat}\text{ ratio}=K_{m\;\left(Rp\;substrate\right)}\;/\;K_{m\;\left(Sp\;substrate\right)}$$

$$k_{cat}/K_{m}\text{ ratio}=\left[k_{cat\;\left(Rp\;substrate\right)}\;/K_{m\;\left(Rp\;substrate\right)}\right]/\;\left[k_{cat\;\left(Sp\;substrate\right)}\;/K_{m\;\left(Sp\;substrate\right)}\right]$$

A *k*_cat_ ratio or *k*_cat_/*K*_m_ ratio greater than one indicates that the enzyme catalyzes the *R*p substrate more efficient than the *S*p substrate, and *vice versa*.

### Monitoring isomer conversion dynamics in enzyme catalysis

The reaction mixture (4 mL) including 60 µM diastereomeric precursor compounds ((*R*p, *S*p)-sof or (*R*p, *S*p)-rem), 3% DMSO, 0.1 mM CoCl_2_, 50 mM CHES (pH 9.0), and appropriate concentration of purified enzyme (W131M and I106A/W131M were used at concentrations of 35.57 nM and 0.72 µM for both substrates, respectively. In contrast, WT-PTE utilized concentrations of 7.99 µM and 15.99 µM for (*R*p, *S*p)-sof and (*R*p, *S*p)-rem, respectively) was incubated at 30°C and aliquoted sample from the reaction for 150 µL every 2 min until 20 min. Then, methanol 600 µL was added to the reaction for reaction termination. The samples were filtered through 0.2 µm before the determination of diastereomer by HPLC using CHIRALPAK IG-U column. The mobile phase A and B were 95% (v/v) methanol and 100% acetonitrile, respectively. The column was performed at 40°C before injecting the sample volume of 50 µL. The elution was performed with 100% of mobile phase A for 3 min and then programmed gradient solvent systems with 0%–35% of mobile phase B for 3–10 min. The HPLC peaks were detected with the wavelength at 270 nm. Hydrolytic activity was determined based on the area under the curve (AUC) formed by the decline in enantiomeric substrate over time (0–20 min) using the formula:

$$\frac{AUC\;of\;isomer\;\left(R\;or\;S\right)\;from\;the\;enzymatic\;reaction\;at\;X\;\min}{AUC\;of\;isomer\;\left(R\;or\;S\right)\;at\;0\;\min}\times 100˙$$

### Synthesis of racemic remdesivir and racemic sofosbuvir precursors

The synthesis of (*R*p, *S*p)-sof was slightly modified from previous reports (Li and Sha, 2008; Cho et al., 2014), whereas the synthesis of (*R*p, *S*p)-rem was adapted from (Warren et al., 2016) (Supplementary data). Briefly, both racemic compounds were prepared through two-step synthesis. For the racemic sofosbuvir precursor, the first step involved synthesizing *L*-alanine isopropyl ester hydrochloride, while for racemic remdesivir precursor, *L*-alanine 2-ethylbutyl ester hydrochloride was used as starting material. These diastereomeric precursors were produced in the second step under controlled conditions of solvent, temperature, stirrer, and reaction time. The resulting product appeared as a light-yellow syrup, comprising a mixture of diastereomeric products (*S*p)- and (*R*p)- in an approximate 1:1 ratio, as confirmed by HPLC and NMR analysis (Supplementary Figures S1–S8).

### Separation of *S*p-diastereomer

The 0.5 g mixture of diastereomeric precursor for sofosbuvir ((*R*p, *S*p)-sof) was dissolved in diisopropyl ether and stirred at 5°C in an ice bath. While stirring, 0.5 mL hexane was added to the solution. The mixture was allowed to stand in a freezer (5°C) for 12 h. The solid product was collected by filtration, washed with a precooled 1:1 mixture of diisopropyl ether and hexane, and dried under vacuum. The obtained compound was recrystallized again under the same condition. Likewise, the 1 g mixture of diastereomeric precursor for remdesivir ((*R*p, *S*p)-rem) was dissolved in 4 mL diisopropyl ether. The solution was gently stirred at room temperature for 5 h. The diastereomers thus formed were filtrated off, washed with a precooled diisopropyl ether, and dried under vacuum (Supplementary data). The pure *S*p-sof and *S*p-rem were confirmed by HPLC and NMR analysis (Supplementary Figures S9–S16).

### Separation of *R*p-diastereomer

The reaction mixture including 250 mg of (*R*p, *S*p)-sof or 25 g of (*R*p, *S*p)-rem, 0.1 mM CoCl_2_, 50 mM HEPES, 10% methanol, and crude extract In1W-PTE (9.39 mg protein for isolation of (*R*p)-sof and 0.56 mg protein for isolation of (*R*p)-rem). The reaction was stirred (150 rpm) at room temperature for 3–7 h, then adding MeOH 100 mL after completion by chiral HPLC monitoring. The crude mixture was extracted with dichloromethane. The combined organic layers were washed with 50 mM HEPES pH 8 (8-10 times) to remove nitrophenol, dried under Na_2_SO_4_, and evaporated (Supplementary data). The pure (*R*p)- product was determined by HPLC and NMR (Supplementary Figures S17–S22).

## Results and discussions

### Computational design of mutations

Enhancing enzyme specificity is to fine-tune enzyme-substrate interactions. In this study, computational structural analysis was employed to modify selectivity of PTE for ProTide synthesis. Synthetic pathways to produce remdesivir and sofosbuvir exist in multiple routes, each involving a complex series of chemical reactions and the use of various solvents (Peifer et al., 2014; Barth et al., 2016; Hu et al., 2022; Kumar Palli et al., 2022). The selected synthetic route used in this study is presented in Figure 2. During the synthesis, racemic mixtures are produced, and subsequent steps are required for isolating and purifying the desired isomer. One of the key chemical intermediates that determine the isomer of these ProTides are 2-ethylbutyl 2-[[(4-nitrophenoxy) (phenoxy) phosphoryl]amino]propanoate ((*R*p, *S*p)-rem, Figure 2B), and propan-2-yl 2-[[(4-nitrophenoxy) (phenoxy)phosphoryl] amino] propanoate ((*R*p, *S*p)-sof, Figure 2B). PTE variants were employed in the precursor isolation step to specifically hydrolyze (*R*p)-isomer therefore the desired (*S*p)-isomer will remain.

**FIGURE 3:**
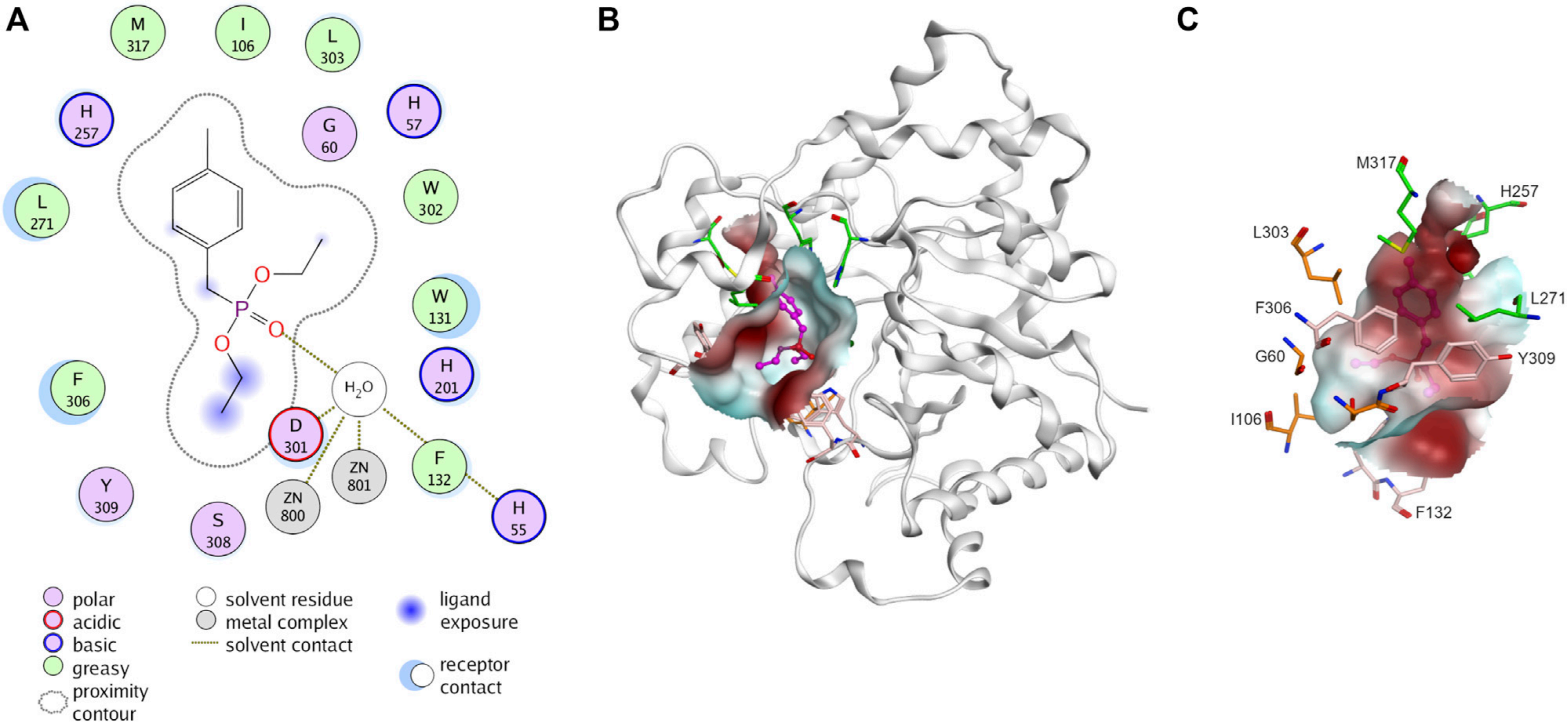
Three-dimensional structure of phosphotriesterase (PTE). **(A)** Interaction of PTE with paraoxon analog, diethyl 4-methylbenzylphosphonate (PDB ID: 1DPM). Receptor and ligand exposures as well as the physiochemical nature of the residues are explained in the color legend. **(B)** Distribution of molecular hydrophobicity at the active site. Ribbon diagrams and molecular surface colored according to hydrophobicity potential are shown. Color scheme: red, hydrophobic; blue, hydrophilic. The structure of ligand is presented by purple balls and sticks model. **(C)** Binding site of PTE. The amino acids at small pocket are presented in orange, large pocket in green and leaving pocket in pink.

PTE is promiscuous on a broad spectrum of diverse substrates. The substrate promiscuity makes it attractive for various applications. PTE enzymes are renowned for their capability to detoxicate organophosphate agents such as paraoxon and parathion (Briseño-Roa et al., 2011). Several studies have shown that it possible to alter PTE specificity for organophosphorus insecticide (Hong and Raushel, 1999; Chen-Goodspeed et al., 2001b; Naqvi et al., 2014; Kronenberg et al., 2024). The PTE have three binding pockets (small, large, and leaving group, Figure 3) (Chen-Goodspeed et al., 2001a; Bigley and Raushel, 2013). The small pocket is composed of G60, I106, L303, and S308. The large pocket and the leaving pocket are highly exposed to solvent. The leaving pocket is composed of hydrophobic side chain of W131, F132, F306 and Y309 whereas the large pocket is defined by H254, H257, L271 and M317 (Figure 3). The hydrolysis of PTE requires two divalent metal ions, commonly zinc ions, for enzyme activity (Hong and Raushel, 1996). These metal ions activate a water molecule, converting it into a hydroxide ion, which then attacks the phosphorus atom of the substrate, leading to the cleavage of the ester bond (Aubert et al., 2004; Bigley and Raushel, 2013; 2019). A product such as p-nitrophenol is released (Figure 2A).

Instead of investigating all possible mutations of amino acids aligned at the binding site, structure-based analysis and design was employed to improve specificity. Molecular docking of each stereoisomer of remdesivir and sofosbuvir precursors were used to specify possible binding poses. The docked poses were selected based on the lowest docking scores with preferred interactions where phosphoryl oxygen should interact with metal ions (Aubert et al., 2004; Bigley and Raushel, 2013; 2019). MD simulations were performed to validate the docked poses. Binding free energies were also calculated to evaluate whether the mutation favored *R*p-rem or *R*p-sof or both (Supplementary data, Supplementary Figures S25, 26; Supplementary Table S3).

To improve selectivity toward (*R*p)-isomer, the mutations were designed either to block the binding of (*S*p)-isomer or to improve the accommodation of (*R*p)-isomer. A small set of PTE variants were generated. Enlarging the size of the small pocket was carried out by substituent I106 with alanine (A) and valine (V) which have smaller side chain but possess hydrophobic side chain akin to isoleucine (I). Similarly, replacement of L303 or S308 with alanine (A) was conducted. To enhance potential pi-interactions, phenylalanine (F) and tyrosine (Y) having benzene ring were introduced at L303 and S308, respectively. W131, an amino acid at leaving group pocket, was substituted with methionine (M) having a smaller hydrophobic side chain. For reshaping the large pocket, substitution of L271 with glutamic acid (E), asparagine (N), or phenylalanine (F) and substitution of H254 with tyrosine (Y) were employed. Moreover, we explored replacing a non-catalytic residue D233 as it was in proximity to the predicted binding of (*R*p)-diastereomer. Although the substitution of D233 was not aimed at enhancing activity toward (*R*p)-diastereomer, it was included to assess the efficacy of rational design. In total, we generated 22 PTE variants in addition to WT-PTE, G60A and In1W. These variants were then employed in screening for stereospecificity.

### Screening for diastereoselective variants

**FIGURE 4:**
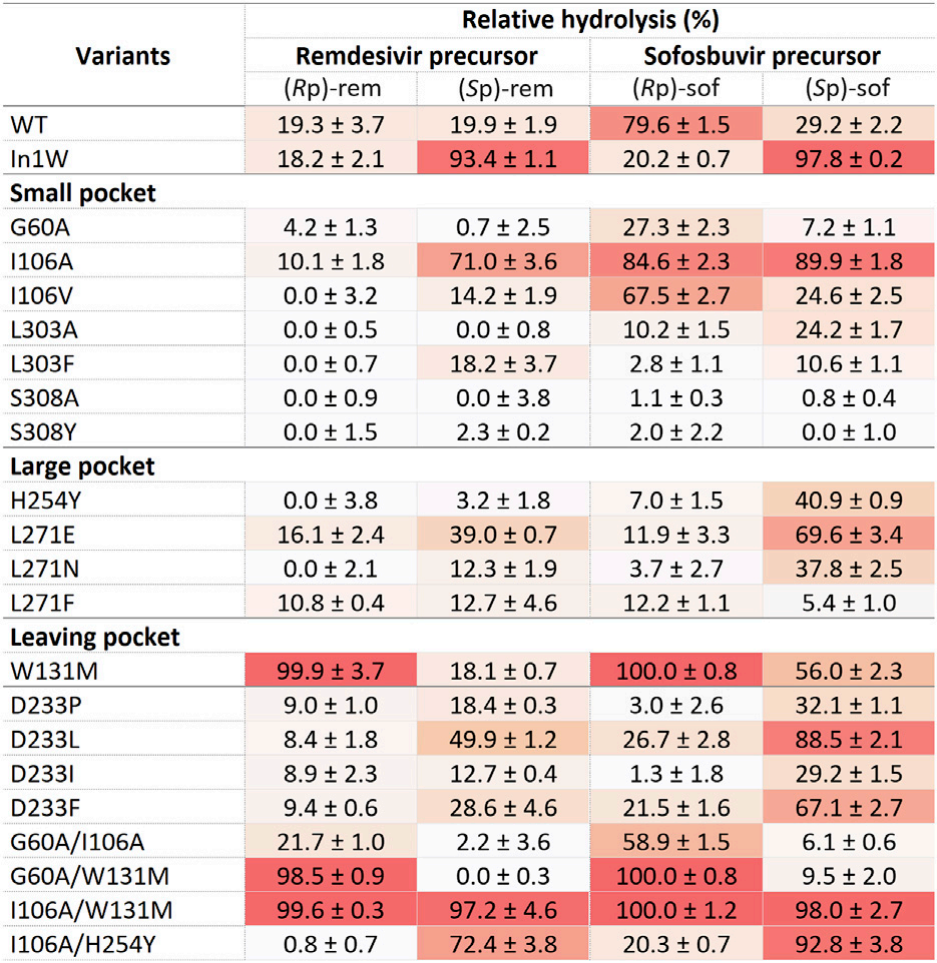
The mutant PTEs screening for determination of stereoselectivity.

Crude enzymes were used in initial screening. Selective hydrolysis was assessed through the relative hydrolysis of individual pure diastereomers of sofosbuvir precursor and remdesivir precursor (Figure 4). The results of In1W and G60A variants agreed with previous report. In1W exhibits a strong preference for (*S*p)-rem and (*S*p)-sof, while G60A was capable of hydrolyzing only (*R*p)-sof and not (*R*p)-rem (Xiang et al., 2019; Bigley et al., 2020). Nevertheless, crude WT enzyme showed higher activity for (*R*p)-sof (79.6%) than the G60A variant. Among mutations in the small pocket, the substitution of I106A resulted in an enhancement of hydrolytic activity for (*S*p)-rem, (*S*p)-sof, and (*R*p)-sof compared to either WT or G60A variant. I106V variant displayed superior hydrolysis of the (*R*p)-sof compared to G60A however, it exhibited diminished activity against (*R*p)-rem. No substitutions of amino acids in the large pocket resulted in hydrolysis improvement of (*R*p)-isomer. Interestingly, W131M mutation in the leaving group pocket, demonstrated nearly absolute 100% relative hydrolysis of both (*R*p)-sof and (*R*p)-rem. The double-mutant G60A/W131M yielded a variant with a higher catalytic efficiency toward (*R*p)-isomer over (*S*p)-isomer for both remdesivir and sofosbuvir precursors. Conversely, the two-point mutation I106A/W131M led to non-selective hydrolysis. The variants exhibiting (*R*p)- selective hydrolysis including G60A, I106A, W131M, G60A/I106A, G60A/W131M, and I106A/W131M were chosen for purification and detailed analysis.

### Enzymatic activity and kinetic characterization of the PTE variants

Kinetic parameters (*K*_m_​, *k*_cat_ and *k*_cat_/*K*_m_) were used to characterize enzyme variants (Table 1). Michaelis constant (*K*_m_​) represents the substrate concentration needed for an enzyme to achieve half of its maximum velocity (Vmax). A high Km value indicates that a large amount of substrate is necessary to saturate the enzyme, implying that the enzyme has low affinity for the substrate. Catalytic activity is indicated by turnover number (*k*_cat_). High *k*_cat_ value suggests a rapid reaction rate. The ratio of (*k*_cat_/*K*_m_) represents the catalytic efficiency. Furthermore, the ratio of the kinetic parameters of (*R*p)- to (*S*p)- (abbreviated *R*p/*S*p) was calculated for comparing substrate stereoselectivity. If the mutant enzyme shows a preference for the *R*p substrate over the Sp substrate, the *K*_m_ ratio (*R*p/*S*p) will be less than 1. Conversely, if *R*p substrate is more efficient, the ratio of *k*_cat_ or *k*_cat_/*K*_m_ for *R*p substrate compared to that for the *S*p substrate will be greater than 1.

**TABLE 1:** Kinetic parameters of purified wild type PTE (WT) and their variants. All values are means of three independent determinations.

Variants	Substrate	*K*_m_ (M)	*K*_*m*_ ratio	Turnover number *k*_cat_ (s^-1^)	k_cat_ ratio	Catalytic efficiency *k*_cat_/*K*_m_ (M^-1^.s^-1^)	k_cat_/K_m_ ratio
			(*Rp/Sp*)		(*Rp/Sp*)		(*Rp/Sp*)
WT	(*R*p)-rem	(1.72 ± 0.17) x 10^−5^	0.56	(4.97 ± 0.10) x 10^−3^	0.46	(2.90 ± 0.26) x 10^2^	0.82
	(*S*p)-rem	(3.05 ± 0.07) x 10^−5^		(1.08 ± 0.01) x 10^−2^		(3.56 ± 0.04) x 10^2^	
	(*R*p)-sof	(1.81 ± 0.16) x 10^−4^	0.67	(1.88 ± 0.24) x 10^−1^	1.57	(1.04 ± 0.04) x 10^3^	2.34
	(*S*p)-sof	(2.71 ± 0.29) x 10^−4^		(1.20 ± 0.11) x 10^−1^		(4.44 ± 0.59) x 10^2^	
G60A	(*R*p)-rem	(4.38 ± 0.11) x 10^−5^	1.66	(1.44 ± 0.24) x 10^−2^	9.03	(3.36 ± 0.45) x 10^2^	5.56
	(*S*p)-rem	(2.64 ± 0.15) x 10^−5^		(1.60 ± 0.09) x 10^−3^		(6.06 ± 0.12) x 10^1^	
	(*R*p)-sof	(4.99 ± 0.62) x 10^−4^	1.43	(1.94 ± 0.09) x 10^−1^	19.26	(3.91 ± 0.36) x 10^2^	13.50
	(*S*p)-sof	(3.48 ± 0.56) x 10^−4^		(1.01 ± 0.14) x 10^−2^		(2.90 ± 0.05) x 10^1^	
I106A	(*R*p)-rem	(7.61 ± 0.15) x 10^−5^	2.72	(6.59 ± 0.13) x 10^−2^	1.01	(8.70 ± 0.86) x 10^2^	0.37
	(*S*p)-rem	(2.80 ± 0.75) x 10^−5^		(6.51 ± 0.98) x 10^−2^		(2.37 ± 0.24) x 10^3^	
	(*R*p)-sof	(6.89 ± 0.74) x 10^−5^	0.16	(4.94 ± 0.19) x 10^−1^	0.60	(7.21 ± 0.48) x 10^3^	3.79
	(*S*p)-sof	(4.33 ± 0.73) x 10^−4^		(8.25 ± 0.15) x 10^−1^		(1.90 ± 0.14) x 10^3^	
W131M	(*R*p)-rem	(1.20 ± 0.20) x 10^−5^	1.54	(0.74 ± 0.01) x 10^1^	291.04	(6.20 ± 0.45) x 10^5^	186.93
	(*S*p)-rem	(7.79 ± 0.13) x 10^−6^		(2.55 ± 0.13) x 10^−2^		(3.32 ± 0.41) x 10^3^	
	(*R*p)-sof	(1.09 ± 0.19) x 10^−4^	0.31	(3.22 ± 0.43) x 10^1^	58.05	(2.97 ± 0.13) x 10^5^	187.28
	(*S*p)-sof	(3.51 ± 0.48) x 10^−4^		(5.54 ± 0.66) x 10^−1^		(1.58 ± 0.10) x 10^3^	
G60A/I106A	(*R*p)-rem	(1.66 ± 0.20) x 10^−4^	24.74	(8.65 ± 0.85) x 10^−2^	111.87	(5.25 ± 0.77) x 10^2^	4.48
	(*S*p)-rem	(6.71 ± 0.11) x 10^−6^		(7.73 ± 0.53) x 10^−4^		(1.17 ± 0.20) x 10^2^	
	(*R*p)-sof	(4.57 ± 0.92) x 10^−4^	0.93	(5.04 ± 0.54) x 10^−1^	30.96	(1.12 ± 0.11) x 10^3^	33.19
	(*S*p)-sof	(4.90 ± 0.12) x 10^−4^		(1.63 ± 0.30) x 10^−2^		(3.37 ± 0.28) x 10^1^	
G60A/W131M	(*R*p)-rem	(5.60 ± 0.96) x 10^−5^	3.74	(0.16 ± 0.01) x 10^1^	189.32	(2.84 ± 0.22) x 10^4^	51.18
	(*S*p)-rem	(1.50 ± 0.11) x 10^−5^		(8.33 ± 0.88) x 10^−3^		(5.55 ± 0.35) x 10^2^	
	(*R*p)-sof	(3.64 ± 0.64) x 10^−4^	0.60	(1.53 ± 0.17) x 10^1^	39.71	(4.24 ± 0.25) x 10^4^	66.36
	(*S*p)-sof	(6.05 ± 0.39) x 10^−4^		(3.86 ± 0.18) x 10^−1^		(6.39 ± 0.11) x 10^2^	
I106A/W131M	(*R*p)-rem	(1.18 ± 0.14) x 10^−5^	0.70	(4.60 ± 0.16) x 10^−1^	233.11	(3.93 ± 0.53) x 10^4^	333.79
	(*S*p)-rem	(1.70 ± 0.22) x 10^−5^		(1.97 ± 0.16) x 10^−3^		(1.18 ± 0.20) x 10^2^	
	(*R*p)-sof	(7.25 ± 0.11) x 10^−5^	0.11	(0.17 ± 0.06) x 10^1^	103.24	(2.38 ± 0.44) x 10^4^	995.58
	(*S*p)-sof	(6.88 ± 0.13) x 10^−4^		(1.64 ± 0.31) x 10^−2^		(2.39 ± 0.07) x 10^1^	

The results (Table 1) showed that both affinity and reaction rate were imperative to considered for optimizing the effectiveness of the enzyme in catalyzing enantioselective process. For example, the lower of *K*_m_ value as well as the *K*_m_ ratio of (*R*p/*S*p) in WT variants indicated the higher affinity toward the (*R*p)-diastereomers of both ProTide precursors compared to theG60A variant. Despite the better affinity, WT enzyme was unable to separate the racemic mixture during the reaction while G60A variant showed that capability (Xiang et al., 2019). This could be explained by the significantly change in the reaction rate. Mutation of G60A resulted in enhancement in the turnover rate specially toward (*R*p)-diastereomers. As a results, the overall catalytic efficiency of G60A toward (*R*p)-diastereomers was better than its effectiveness toward (*S*p)-diastereomers. Most PTE variants except G60A exhibited improved affinity toward (*R*p)-diastereomer as indicated by the lower *K*_m_​ ratio of (*R*p/*S*p). However, only the I106A/W131M showed a preference for both (*R*p)-rem (*K*_m_​ ratio = 0.70) and (*R*p)-sof (*K*_m_​ ratio = 0.11). Remarkably elevated turnover numbers were detected in variant W131M and I106A/W131M (Table 1). In W131M variant, the turnover number ratio toward (*R*p/*S*p)-rem (291.04) was highest compared to the relative ratio of other variants. Interestingly, while the introduction of a single point mutation at I106A showed marginal enhancement of turnover rate compared to W131M, the synergistic effect of combining I106A and W131M led to significant improvement. The catalytic efficiency ratio of (*R*p/*S*p)-rem, increased by 333.79-fold, while in (*R*p/*S*p)-sof, it surged to 995.58 (Table 1). The results suggested the W131M and I106A/W131M variants have potential for utilization in stereoselective hydrolysis.

**FIGURE 5:**
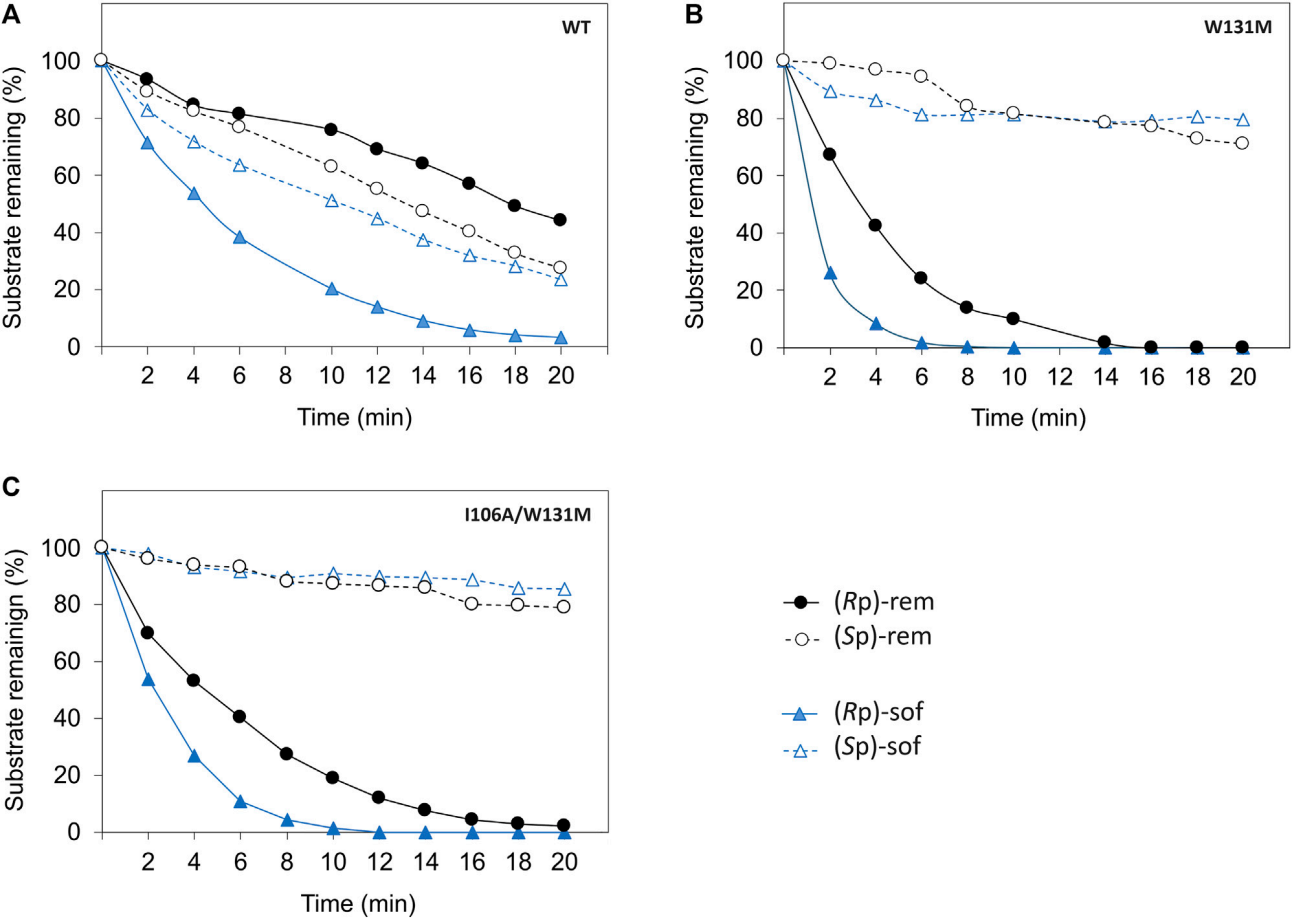
Time course of enzymatic hydrolysis of the ProTide precursors with different PTE variants including **(A)** wild type PTE, **(B)** W131M, and **(C)** I106A/W131M. The reactions were carried out as follows. The Enzymes were incubated at 30°C with 60 µM of each diastereomeric substrate in a reaction mixture at optimized concentrations determined from prior experimentation. WT-PTE was used at concentrations of 7.99 µM for (*R*p, *S*p)-sof and 15.99 µM for (*R*p, *S*p)-rem, while mutant enzymes (W131M and I106A/W131M) were used at 35.57 nM and 0.72 µM for both precursors, respectively. Aliquots were taken every 2 min, quenched with methanol, and analyzed by HPLC using a CHIRALPAK IG-U column with gradient elution. Detection was performed at 270 nm, and the remaining substrates percentage were determined from the area under the curve.

As hydrolysis time is also an important factor for industrial use of a biocatalyst, we further investigated time course of hydrolysis for the preparation of pure diastereomer using W131M and I106A/W131M in comparison to WT (Figure 5). In WT, the kinetic properties (Table 1) showed a greater catalytic efficiency for sofosbuvir precursors compared to remdesivir precursors and a higher preference for (*R*p)-diastereomer than (*S*p)-diastereomer. Nevertheless, WT could not completely hydrolyze (*R*p)-sof within 20 min of hydrolysis. At 20 min, the remaining (*R*p)-sof was 3%, while the remaining (*S*p)-sof was 23% (Figure 5A). On the other hand, W131M showed complete hydrolysis of (*R*p)-sof and (*R*p)-rem within 8 min and 16 min, respectively (Figure 5B). At the same time, it retained a high amount of the desired isomer, with 81% of (*S*p)-sof at 8 min and 77% of (*R*p)-rem at 16 min.

For I106A/W131M variant, the enzyme could completely hydrolyze the (*R*p)-sof within 12 min while the remaining of the pure (*S*p)-sof was over 90% (Figure 5C). However, I106A/W131M variant could not completely hydrolyze (*R*p)-rem within 20 min. At 20 min, hydrolysis of I106A/W131M left 2% of (*R*p)-rem remaining while 79% of (*S*p)-rem was retained. The rapid hydrolysis achieved in a short period (<20 min) effectively depleted unwanted diastereomers, justifying the expediency of preparing pure diastereomers. These results imply that the enzymes developed in this study could facilitate the development of cost-effective bioprocesses for preparing ProTide drugs in a batch system.

PTE exhibits proficiency in hydrolyzing diverse substrates, notable variations arise in their catalytic efficiencies, often attributed to differences in the size of the binding pocket (Chen-Goodspeed et al., 2001b; Bigley and Raushel, 2013; 2019). Previous studies on the synthesis of sofosbuvir and remdesivir focused on selective degradation of *S*p-isomers (Xiang et al., 2019; Bigley et al., 2020). PTE variants that were originally obtained from catalytic activity screening for other substrates were employed to screen stereoselectivity toward phosphonate substrates. These variants included G60A (Chen-Goodspeed et al., 2001a) and I106G/F132G/H257Y (Chen-Goodspeed et al., 2001b), H257Y/L303T (Tsai et al., 2010), and In1W (Bigley et al., 2013). In this study, new PTE variants were initially selected based on computational prediction of enzyme-substrate interactions to improve enantioselectivity toward *R*p-isomers. Subsequently, experimental screening and kinetic study were conducted. Of the PTE variants tested, W131M and I106A/W131M showed high diastereospecificity which could selectively degrade (*R*p)-diastereomer while preserve (*S*p)-diastereomer. These PTE variants have good potential for producing stereoisomericallly pure sofosbuvir, remdesivir as well as other ProTides.

The kinetic analysis showed an aspect that should be considered for future development. Although the affinity of the variants were improved as designing, the kinetic data suggested that flexibility had a major role in enzyme selectivity of PTE. High affinity for the ligand may resulted in decreasing of turnover speed (Kari et al., 2021) which could consequently resulted in reduction of catalytic efficiency and substrate selectivity. Moving forward, further studies leveraging advanced computational modeling and turnover number prediction hold the promise of unlocking new strategies to fine-tune biocatalysts for enhanced selectivity and efficiency in diverse applications. By navigating this intricate balance, biocatalyst designers can pave the way for transformative advancements in biotechnology and pharmaceutical synthesis.

## Data Availability

The datasets presented in this study can be found in online repositories. The names of the repository/repositories and accession number(s) can be found in the article/Supplementary Material.

## References

[B1] Adams J. P. Brown M. J. B. Diaz-Rodriguez A. Lloyd R. C. Roiban G.-D. (2019). Biocatalysis: a pharma perspective. Adv. Synthesis Catal. 361, 2421–2432. 10.1002/adsc.201900424

[B2] Alcántara A. R. Domínguez De María P. Littlechild J. A. Schürmann M. Sheldon R. A. Wohlgemuth R. (2022). Biocatalysis as key to sustainable industrial chemistry. ChemSusChem 15, e202200709. 10.1002/cssc.202200709 35445559

[B3] Aubert S. D. Li Y. Raushel F. M. (2004). Mechanism for the hydrolysis of organophosphates by the bacterial phosphotriesterase. Biochemistry 43, 5707–5715. 10.1021/bi0497805 15134445

[B4] Barth R. Rose C. A. Schöne O. (2016). “Synthetic routes to sofosbuvir,” in Synthesis of heterocycles in contemporary medicinal chemistry. Editor Časar Z. (Cham: Springer International Publishing), 51–88.

[B5] Berman H. M. Westbrook J. Feng Z. Gilliland G. Bhat T. N. Weissig H. (2000). The protein data bank. Nucleic Acids Res. 28, 235–242. 10.1093/nar/28.1.235 10592235 PMC102472

[B6] Bigley A. N. Narindoshvili T. Raushel F. M. (2020). A chemoenzymatic synthesis of the (RP)-Isomer of the antiviral prodrug remdesivir. Biochemistry 59, 3038–3043. 10.1021/acs.biochem.0c00591 32786401 PMC7418565

[B7] Bigley A. N. Raushel F. M. (2013). Catalytic mechanisms for phosphotriesterases. Biochim. Biophys. Acta 1834, 443–453. 10.1016/j.bbapap.2012.04.004 22561533 PMC3421070

[B8] Bigley A. N. Raushel F. M. (2019). The evolution of phosphotriesterase for decontamination and detoxification of organophosphorus chemical warfare agents. Chem. Biol. Interact. 308, 80–88. 10.1016/j.cbi.2019.05.023 31100274 PMC6622166

[B9] Bigley A. N. Xu C. Henderson T. J. Harvey S. P. Raushel F. M. (2013). Enzymatic neutralization of the chemical warfare agent VX: evolution of phosphotriesterase for phosphorothiolate hydrolysis. J. Am. Chem. Soc. 135, 10426–10432. 10.1021/ja402832z 23789980 PMC3747228

[B10] Briseño-Roa L. Oliynyk Z. Timperley C. M. Griffiths A. D. Fersht A. R. (2011). Highest paraoxonase turnover rate found in a bacterial phosphotriesterase variant. Protein Eng. Des. Sel. 24, 209–211. 10.1093/protein/gzq046 20650962

[B11] Chemical Computing Group Ulc (2023). Molecular operating environment (MOE), 2022.02”. 1010 sherbooke st. West, suite #910. Montreal, QC, Canada: MOE. H3A 2R7.

[B12] Chen-Goodspeed M. Sogorb M. A. Wu F. Hong S.-B. Raushel F. M. (2001a). Structural determinants of the substrate and stereochemical specificity of phosphotriesterase. Biochemistry 40, 1325–1331. 10.1021/bi001548l 11170459

[B13] Chen-Goodspeed M. Sogorb M. A. Wu F. Raushel F. M. (2001b). Enhancement, relaxation, and reversal of the stereoselectivity for phosphotriesterase by rational evolution of active site residues. Biochemistry 40, 1332–1339. 10.1021/bi001549d 11170460

[B14] Cho A. Zhang L. Xu J. Lee R. Butler T. Metobo S. (2014). Discovery of the first C-nucleoside HCV polymerase inhibitor (GS-6620) with demonstrated antiviral response in HCV infected patients. J. Med. Chem. 57, 1812–1825. 10.1021/jm400201a 23547794

[B15] Dousson C. B. (2018). Current and future use of nucleo(s)tide prodrugs in the treatment of hepatitis C virus infection. Antivir. Chem. Chemother. 26, 204020661875643. 10.1177/2040206618756430 PMC5890546 29463095

[B16] Fda (1992). FDA'S policy statement for the development of new stereoisomeric drugs. Chirality 4, 338–340. 10.1002/chir.530040513 1354468

[B17] Hong S.-B. Raushel F. M. (1996). Metal−Substrate interactions facilitate the catalytic activity of the bacterial phosphotriesterase. Biochemistry 35, 10904–10912. 10.1021/bi960663m 8718883

[B18] Hong S.-B. Raushel F. M. (1999). Stereochemical constraints on the substrate specificity of phosphotriesterase. Biochemistry 38, 1159–1165. 10.1021/bi982204m 9930975

[B19] Hu T. Zhu F. Xiang L. Shen J. Xie Y. Aisa H. A. (2022). Practical and highly efficient synthesis of remdesivir from GS-441524. ACS Omega 7, 27516–27522. 10.1021/acsomega.2c02835 35967033 PMC9366944

[B20] Kari J. Molina G. A. Schaller K. S. Schiano-Di-Cola C. Christensen S. J. Badino S. F. (2021). Physical constraints and functional plasticity of cellulases. Nat. Commun. 12, 3847. 10.1038/s41467-021-24075-y 34158485 PMC8219668

[B21] Kronenberg J. Chu S. Olsen A. Britton D. Halvorsen L. Guo S. (2024). Computational design of phosphotriesterase improves V-agent degradation efficiency. ChemistryOpen 13, e202300263. 10.1002/open.202300263 38426687 PMC11230934

[B22] Kumar Palli K. Ghosh P. Krishna Avula S. Sridhara Shanmukha Rao B. Patil A. D. Ghosh S. (2022). Total synthesis of remdesivir. Tetrahedron Lett. 88, 153590. 10.1016/j.tetlet.2021.153590 34908617 PMC8656175

[B23] Lewis R. D. France S. P. Martinez C. A. (2023). Emerging technologies for biocatalysis in the pharmaceutical industry. ACS Catal. 13, 5571–5577. 10.1021/acscatal.3c00812

[B24] Li J. Sha Y. (2008). A convenient synthesis of amino acid methyl esters. Molecules 13, 1111–1119. 10.3390/molecules13051111 18560331 PMC6245331

[B25] Liang C. Tian L. Liu Y. Hui N. Qiao G. Li H. (2020). A promising antiviral candidate drug for the COVID-19 pandemic: a mini-review of remdesivir. Eur. J. Med. Chem. 201, 112527. 10.1016/j.ejmech.2020.112527 32563812 PMC7834743

[B26] Mehellou Y. Rattan H. S. Balzarini J. (2018). The ProTide prodrug technology: from the concept to the clinic. J. Med. Chem. 61, 2211–2226. 10.1021/acs.jmedchem.7b00734 28792763 PMC7075648

[B27] Naqvi T. Warden A. C. French N. Sugrue E. Carr P. D. Jackson C. J. (2014). A 5000-fold increase in the specificity of a bacterial phosphotriesterase for malathion through combinatorial active site mutagenesis. PLOS ONE 9, e94177. 10.1371/journal.pone.0094177 24721933 PMC3983157

[B28] Peifer M. Berger R. Shurtleff V. W. Conrad J. C. Macmillan D. W. C. (2014). A general and enantioselective approach to pentoses: a rapid synthesis of PSI-6130, the nucleoside core of sofosbuvir. J. Am. Chem. Soc. 136, 5900–5903. 10.1021/ja502205q 24670208 PMC4210058

[B29] Raran-Kurussi S. Waugh D. S. (2012). The ability to enhance the solubility of its fusion partners is an intrinsic property of maltose-binding protein but their folding is either spontaneous or chaperone-mediated. PLOS ONE 7, e49589. 10.1371/journal.pone.0049589 23166722 PMC3500312

[B30] Roodveldt C. Tawfik D. S. (2005). Directed evolution of phosphotriesterase from Pseudomonas diminuta for heterologous expression in *Escherichia coli* results in stabilization of the metal-free state. Protein Eng. Des. Sel. 18, 51–58. 10.1093/protein/gzi005 15790580

[B31] Rossino G. Robescu M. S. Licastro E. Tedesco C. Martello I. Maffei L. (2022). Biocatalysis: a smart and green tool for the preparation of chiral drugs. Chirality 34, 1403–1418. 10.1002/chir.23498 35929567 PMC9805200

[B32] Rougeot C. Hein J. E. (2015). Application of continuous preferential crystallization to efficiently access enantiopure chemicals. Org. Process Res. Dev. 19, 1809–1819. 10.1021/acs.oprd.5b00141

[B33] Sabat N. Ouarti A. Migianu-Griffoni E. Lecouvey M. Ferraris O. Gallier F. (2022). Synthesis, antiviral and antitumor activities investigations of a series of Ribavirin C-nucleoside analogue prodrugs. Bioorg. Chem. 122, 105723. 10.1016/j.bioorg.2022.105723 35278778

[B34] Sheldon R. A. Brady D. Bode M. L. (2020). The Hitchhiker's guide to biocatalysis: recent advances in the use of enzymes in organic synthesis. Chem. Sci. 11, 2587–2605. 10.1039/c9sc05746c 32206264 PMC7069372

[B35] Slusarczyk M. Serpi M. Pertusati F. (2018). Phosphoramidates and phosphonamidates (ProTides) with antiviral activity. Antivir. Chem. Chemother. 26, 204020661877524. 10.1177/2040206618775243 PMC5971382 29792071

[B36] Sofia M. J. Bao D. Chang W. Du J. Nagarathnam D. Rachakonda S. (2010). Discovery of a β-d-2′-Deoxy-2′-α-fluoro-2′-β-*C*-methyluridine nucleotide prodrug (PSI-7977) for the treatment of hepatitis C virus. J. Med. Chem. 53, 7202–7218. 10.1021/jm100863x 20845908

[B37] Sui J. Wang N. Wang J. Huang X. Wang T. Zhou L. (2023). Strategies for chiral separation: from racemate to enantiomer. Chem. Sci. 14, 11955–12003. 10.1039/d3sc01630g 37969602 PMC10631238

[B38] Tsai P.-C. Bigley A. Li Y. Ghanem E. Cadieux C. L. Kasten S. A. (2010). Stereoselective hydrolysis of organophosphate nerve agents by the bacterial phosphotriesterase. Biochemistry 49, 7978–7987. 10.1021/bi101056m 20701311 PMC2945820

[B39] Vanhooke J. L. Benning M. M. Raushel F. M. Holden H. M. (1996). Three-dimensional structure of the zinc-containing phosphotriesterase with the bound substrate analog diethyl 4-methylbenzylphosphonate. Biochemistry 35, 6020–6025. 10.1021/bi960325l 8634243

[B40] Warren T. K. Jordan R. Lo M. K. Ray A. S. Mackman R. L. Soloveva V. (2016). Therapeutic efficacy of the small molecule GS-5734 against Ebola virus in rhesus monkeys. Nature 531, 381–385. 10.1038/nature17180 26934220 PMC5551389

[B41] Xiang D. F. Bigley A. N. Desormeaux E. Narindoshvili T. Raushel F. M. (2019). Enzyme-catalyzed kinetic resolution of chiral precursors to antiviral prodrugs. Biochemistry 58, 3204–3211. 10.1021/acs.biochem.9b00530 31268686 PMC6822272

