# Supplementary material for: Improving stereoselectivity of phosphotriesterase (PTE) for kinetic resolution of chiral phosphates
Source: Front Bioeng Biotechnol. 2024 Jul 30;12:1446566. doi: 10.3389/fbioe.2024.1446566 (PMC11319162; doi:10.3389/fbioe.2024.1446566)
Supplement: Supplementary file 1 [file DataSheet1.docx]

Supplementary Material

# Supplementary Data

*

*

**Scheme 1**. Synthesis route of diastereomeric precursors of Sofosbuvir (2) and Remdesivir (4).

**Synthesis of diastereomeric precursor for Sofosbuvir [(*R*p/*S*p)-sof]**

*Step 1: synthesis of L-alanine isopropyl ester hydrochloride (****1****)*

The suspension of *L*-alanine (20 g, 224.5 mmol) and Trimethylsilyl chloride (TMSCl) (120 mL, 945.5 mmol) in isopropyl alcohol (200 mL, 2,616 mmol) was heated to reflux for 4 h. The reaction was cooled to room temperature, evaporated, and dried under vacuum to obtain the product as a white solid (36 g, 96% yield).; ^1^H NMR (CDCl_3_, 500 MHz): δ (ppm) 8.5 (bs, 3H), 5.06 (m, 1H), 4.15 (dd, *J* = 12.02, 6.1 Hz, 1H), 1.65 (d, *J* = 6.3 Hz, 3H), 1.2 (t, *J* = 5.94 Hz, 6H).

*Step 2: synthesis of isopropyl ((4-nitrophenoxy)(phenoxy)phosphoryl)-L-alaninate (****2****)*

The solution of *L*-alanine isopropyl ester hydrochloride (5 g, 29.8 mmol) in DCM (50 mL) was added to phenyl phosphorodichloridate (4.45 mL, 29.8 mmol) at -20°C. Then, triethylamine (8.3 mL, 59.65 mmol) was added dropwise to the mixture over a period of 30 min. The reaction was stirred for 5.5 h. After the reaction was almost complete (by ^31^P NMR), the reaction was cooled to 0°C and 4-nitrophenol (5.6 g, 40.2 mmol) was added to the reaction. Subsequently, triethylamine (6.22 mL, 44.7 mmol) was added dropwise to the reaction over a period of 30 min. The reaction was allowed to warm to room temperature and stirred for 18 h. The reaction was evaporated and extracted with EtOAc (3 x 20 mL). The combined organic layers were washed with 1 M NaOH (5 x 20 mL) and brine solution (50 mL), dried under Na_2_SO_4_, and evaporated to obtain a light yellow syrup of product (7.1 g, 58% yield) as a mixture of diastereomeric product *S*p and *R*p in about 1:1 ratio (determined by HPLC).; ^1^H NMR (DMSO-*d_6_*, 500 MHz): δ (ppm) 8.33 – 8.28 (m, 2H), 7.54 – 7.44 (m, 2H), 7.44 – 7.38 (m, 2H), 7.30 – 7.20 (m, 3H), 6.72 – 6.63 (m, 1H), 4.89 – 4.78 (m, 1H), 3.98 – 3.88 (m, 1H), 1.23 – 1.20 (m, 3H), 1.14 – 1.08 (m, 6H). ^31^P NMR (DMSO-*d_6_*, 202 MHz): δ (ppm) -1.41 (*S*p), -1.57 (*R*p). HPLC R_t_ = 4.84 min (*R*p), 6.13 min (*S*p).

**Synthesis of diastereomeric precursor for Remdesivir [(*R*p/*S*p)-sof]**

*Step 1: synthesis of L-alanine 2-ethylbutyl ester (****3****)*

The suspension of *L*-alanine (10.86 g, 121.9 mmol) and *p*-toluenesulfonic acid monohydrate (p-TsOH·H_2_O) (23.18 g, 121.9 mmol) in toluene (50 mL) was add 2-ethyl-1-butanol (10 mL, 81.24 mmol). The reaction was heated to reflux for 2 h. The reaction was cooled to room temperature and quenched with 1 NaOH (20 mL). Then, the mixture was extracted with EtOAc (3 x 20 mL). The combined organic layers were dried under Na_2_SO_4_ and evaporated to obtain the product as a colorless oil (13.9 g, 99% yield).; ^1^H NMR (CDCl_3_, 500 MHz): δ (ppm) 4.18 –4.07 (m, 3H), 1.57-1.54 (m, 1H), 1.54 –1.48 (m, 3H), 1.40 – 1.32 (m, 4H), 0.92 – 0.89 (m, 4H).

*Step 2: synthesis of 2-ethylbutyl ((4-nitrophenoxy)(phenoxy)phosphoryl)-L-alaninate (****4****)*

The solution of *L*-alanine 2-ethylbutyl ester hydrochloride (7 g, 40.40 mmol) in DCM (50 mL) was added to phenyl phosphorodichloridate (6.05 mL, 40.40 mmol) at -78°C. Then, triethylamine (5.60 mL, 40.40 mmol) was added dropwise to the mixture. The reaction was stirred for 3 h. After the reaction was almost complete (by ^31^P NMR), the reaction was cooled to 0°C and 4-nitrophenol (5.11 g, 36.73 mmol) was added to the reaction. Subsequently, triethylamine (5.6 mL, 40.40 mmol) was added dropwise to the reaction. The reaction was allowed to warm to room temperature and stirred for 3 h. The reaction was evaporated and extracted with EtOAc (3 x 20 mL). The combined organic layers were dried under Na_2_SO_4_ and evaporated to obtain a light yellow syrup of product (14.56 g, 80% yield) as a mixture of diastereomeric product *S*p and *R*p in about 1:1 ratio (determined by HPLC).; ^1^H NMR (DMSO-*d_6_*, 500 MHz): δ (ppm) 8.32 – 8.28 (m, 2H), 7.53 – 7.44 (m, 2H), 7.43 – 7.38 (m, 2H), 7.29 – 7.20 (m, 3H), 6.74 – 6.66 (m, 1H), 4.05 – 3.95 (m, 1H), 3.95 – 3.87 (m, 2H), 1.43 – 1.35 (m, 1H), 1.30 – 1.18 (m, 7H), 0.79 (t, *J* = 7.4 Hz, 6H). ^31^P NMR (MeOD, 202 MHz): δ (ppm) -1.40 (*S*p), -1.63 (*R*p). HPLC R_t_ = 9.37 min (*R*p), 10.89 min (*S*p).

**Recrystallization of *S*p-Sofosbuvir precursor [(*S*p)-sof]**

The mixture of diastereomeric product (0.5 g) was dissolved in diisopropyl ether (2.5 mL, 5 mL/g) and stirred at 5°C in an ice bath. While stirring, hexane (0.5 mL) was added to the solution. The mixture was then added a small amount of seeds of the *S*p diastereomer and gently stirred at 5°C until the white solid appeared. Then the mixture was allowed to stand in a freezer (5°C) for 12 h. The solid product was collected by filtration, washed with a precooled 1:1 mixture of diisopropyl ether and hexane, and dried under vacuum to obtain the white solid (0.0733 g, 15% recovery) as a 92:8 ratio of diastereomers *S*p and *R*p (determined by ^31^P NMR). The product was recrystallized from diisopropyl ether (0.59 mL, 8 mL/g). The solution was gently stirred at 5°C until the white solid appeared. Then the mixture was allowed to stand in a freezer (5°C) for 12 h. The solid product was collected by filtration, washed with a precooled 1:1 mixture of diisopropyl ether and hexane, and dried under vacuum to obtain the white fluffy solid (0.0270 g, 37% recovery) with the 99.3% (*S*p)-Sofosbuvir precursor, (*S*p)-sof (determined by HPLC). ^1^H NMR (MeOD, 500 MHz): δ (ppm) 8.32 – 8.27 (m, 2H), 7.51 – 7.47 (m, 2H), 7.42 – 7.36 (m, 2H), 7.28 – 7.21 (m, 3H), 4.98 – 4.90 (m, 1H), 4.05 – 3.97 (m, 1H), 1.31 (d, *J* = 7.1 Hz, 3H), 1.21 – 1.17 (m, 6H). ^13^C NMR (MeOD, 125 MHz): δ (ppm). ^31^P NMR (DMSO-*d_6_*, 202 MHz): δ (ppm) -1.41 (*S*p). HPLC R_t_ = 6.13 min (*S*p).

**Recrystallization of *S*p-Remdesivir precursor [(*S*p)-rem]**

The mixture of diastereomeric product (1 g) was dissolved in diisopropyl ether (4 mL, 4 mL/g). The solution was gently stirred at room temperature for 5 h. The white solid thus formed was filtrated off, washed with a precooled diisopropyl ether, and dried under vacuum to obtain the white solid (0.45 g, 45% recovery) with the 98.4% (*S*p)-Remdesivir precursor [(*S*p)-rem] (determined by HPLC). ^1^H NMR (DMSO-*d_6_*, 500 MHz): δ (ppm) 8.32 – 8.25 (m, 2H), 7.50 (d, J = 8.8 Hz, 2H), 7.43 – 7.37 (m, 2H), 7.26 – 7.19 (m, 3H), 6.73 – 6.66 (m, 1H), 4.07 – 3.96 (m, 1H), 3.92 (d, J = 5.6 Hz, 2H), 1.43 – 1.35 (m, 1H), 1.30 – 1.17 (m, 7H), 0.79 (t, J = 7.4 Hz, 6H). ^13^C NMR (DMSO-*d_6_*, 125 MHz): δ (ppm) 172.78, 172.73, 155.58, 155.52, 150.18, 150.11, 144.06, 129.80, 125.74, 125.15, 121.06, 121.00, 120.19, 120.15, 66.19, 50.00, 39.65, 22.50, 22.48, 19.68, 19.61, 10.73, 10.71. ^31^P NMR (DMSO-*d_6_*, 202 MHz): δ (ppm) -1.40 (*S*p). HPLC R_t_ = 10.84 min (*S*p).

**Isolation of *R*p-Sofosbuvir precursor [(*R*p)-sof] using In1W-PTE**

The solution of the racemic compound in MeOH 10 mg/mL (250 mg, 25 mL) was added to the mixture of 0.1 mM CoCl_2_ (1 mM CoCl_2_ stock solution, 25 mL, 25 µmol), 50 mM HEPES (250 mM HEPES pH 8 stock solution, 50 mL), MeOH (50 mL), and water (100 mL) (250 mL total reaction volume). Then 4.23 mg/mL crude extract In1W-PTE (2.22 mL, 9.39 mg) was added to the mixture. The reaction to was stirred (150 rpm) at room temperature. The reaction was monitored by ^1^H NMR. After 3 h, the reaction was quenched by adding 100 mL MeOH and evaporated to remove MeOH. The crude product was extracted with DCM (3 x 100 mL). The combined organic layers were washed with 50 mM HEPES pH 8 (8-10 times) to remove nitrophenol, dried under Na_2_SO_4_, and evaporated to obtain the yellow oil of the compound (81.5 mg, 65% yield) with the 99.5% diastereomerically pure *R*p (determined by HPLC). ^1^H NMR (MeOD, 500 MHz): δ (ppm) 8.32 – 8.27 (m, 2H), 7.47 – 7.43 (m, 2H), 7.42 – 7.37 (m, 2H), 7.31 – 7.27 (m, 2H), 7.26 – 7.22 (m, 1H), 4.97 – 4.90 (m, 1H), 4.05 – 3.97 (m, 1H), 1.33 (d, J = 7.1 Hz, 3H), 1.21 – 1.16 (m, 6H). ^31^P NMR (MeOD, 202 MHz): δ (ppm) -1.66 (*R*p). HPLC R_t_ = 4.50 min (*R*p).

**Isolation of *R*p-Remdesivir precursor [(*R*p)-rem] using In1W-PTE**

The solution of the racemic compound in MeOH 10 mg/mL (50 mg, 5 mL) was added to the mixture of 0.1 mM CoCl_2_ (1 mM CoCl_2_ stock solution, 25 mL, 25 µmol), 50 mM HEPES (250 mM HEPES pH 8 stock solution, 50 mL), MeOH (70 mL), and water (100 mL) (250 mL total reaction volume). Then 4.23 mg/mL crude extract In1W-PTE (132.9 µL, 0.56 mg) was added to the mixture. The reaction was stirred (100 rpm) at room temperature. The reaction was monitored by ^1^H NMR. At 3 h, The 4.23 mg/mL crude extract In1W-PTE (132.9 µL, 0.56 mg) was added to the reaction. After 7 h, the reaction was quenched by adding 100 mL MeOH and evaporated to remove MeOH. The crude product was extracted with DCM (3 x 20 mL). The combined organic layers were washed with 50 mM HEPES pH 8 (8-10 times) to remove nitrophenol, dried under Na_2_SO_4_, and evaporated to obtain the yellow oil of the compound (21.6 mg, 86% yield) with the 96% diastereomerically pure *R*p (determined by HPLC). ^1^H NMR (MeOD, 500 MHz): δ (ppm) 8.31 – 8.27 (m, 2H), 7.44 (d, J = 8.7 Hz, 2H), 7.42 – 7.37 (m, 2H), 7.31 – 7.27 (m, 2H), 7.26 – 7.22 (m, 1H), 4.11 – 4.03 (m, 1H), 4.01 (d, J = 5.7 Hz, 2H), 1.49 – 1.42 (m, 1H), 1.37 – 1.30 (m, 7H), 0.87 (t, J = 7.4 Hz, 6H). ^31^P NMR (MeOD, 202 MHz): δ (ppm) -1.75 (*R*p). HPLC R_t_ = 9.09 min (*R*p).

**Molecular dynamics (MD) simulation**

The AMBER 22 software package was used for MD analysis (D.A. Case, 2022). The topology and coordinate data were produced with Antechamber and LEap modules. The force field of FF14SB was employed for parameterization (Maier et al., 2015). The protein-ligand complex structures were solvated with TIP3P water molecules and neutralized with Cl^-^ ions. The minimization of the system was conducted to alleviated steric clashed and ensure an energetically favorable starting conformation. Initial minimization was conducted with 3000 cycles, using the steepest descent algorithm for the first 1000 cycles followed by the conjugate gradient algorithm. Periodic boundary conditions were applied with a non-bonded cutoff of 10 Å. Position restraints were applied to the complex, with a harmonic restraint of 5 kcal/mol/Å². After that minimization without positional restrains were applied. A total of 2000 cycles, with the first 1000 cycles using the steepest descent algorithm and the remaining cycles using the conjugate gradient algorithm, were conducted. Periodic boundary conditions were applied, with a non-bonded cutoff of 10 Å. Then, the systems were heated from 0 to 300 K using SHAKE algorithm and the Langevin thermostat (Wu and Brooks, 2011) within 100 ps with weak restraints (2.0 kcal/mol/Å^2^). The simulation time was set at 50 ns. The root-mean-square deviation (RMSD) was computed to structure stability. The intermolecular binding free energies were estimated using the Generalized Born accessibility algorithms (MMGB-SA)  by processing 1000 frames of last 2ns.

# Supplementary Figures and Tables

## Supplementary Figures


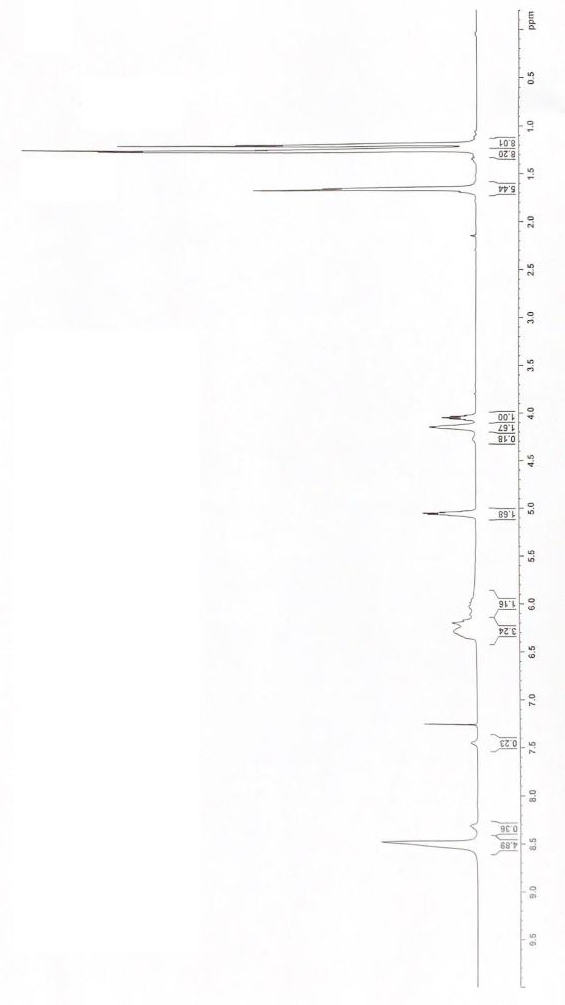


**Supplementary Figure 3.** ^1^H NMR of *L*-alanine isopropyl ester hydrochloride.

**Supplementary Figure 4.** ^1^H NMR of isopropyl ((4-nitrophenoxy)(phenoxy)phosphoryl)-*L*-alaninate.

**Supplementary Figure 5.** ^31^P NMR of isopropyl ((4-nitrophenoxy)(phenoxy)phosphoryl)-*L*-alaninate.


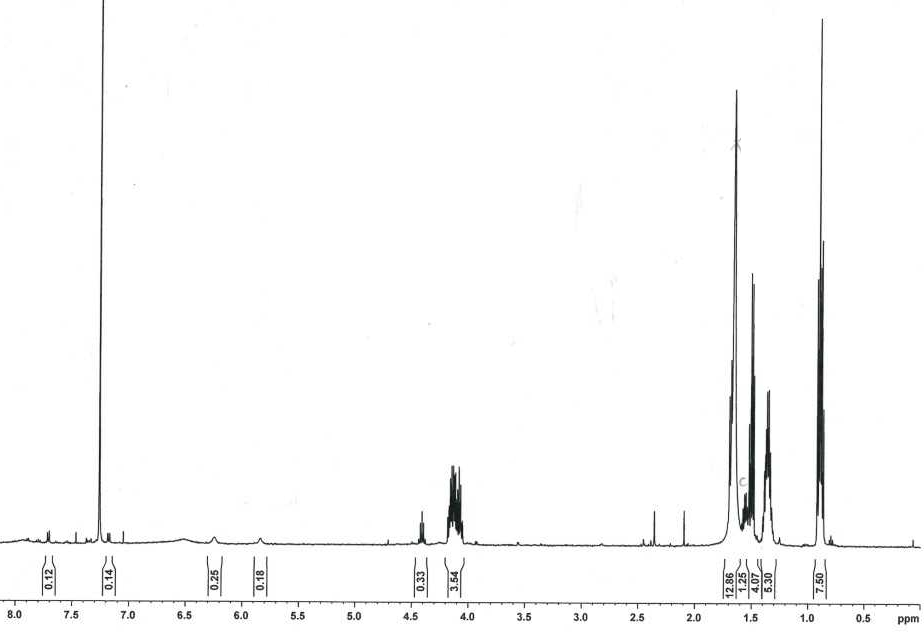


**Supplementary Figure 6.** ^1^H NMR of *L*-alanine 2-ethylbutyl ester.

**Supplementary Figure 7.** ^1^H NMR of 2-ethylbutyl ((4-nitrophenoxy)(phenoxy)phosphoryl)-*L*-alaninate.

**Supplementary Figure 8.** ^31^P NMR of 2-ethylbutyl ((4-nitrophenoxy)(phenoxy)phosphoryl)-*L*-alaninate.


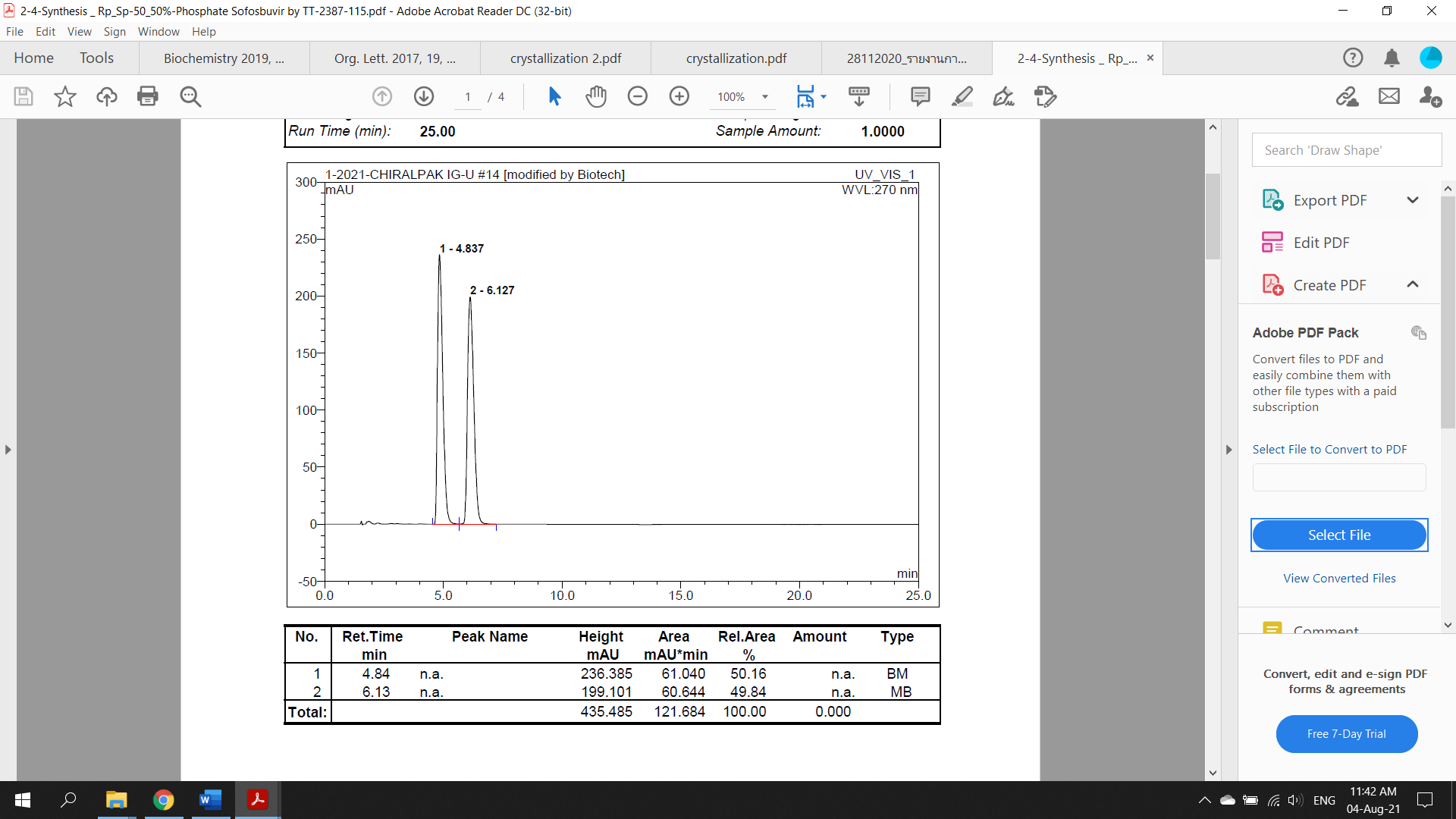


**Supplementary Figure 9.** HPLC of isopropyl ((4-nitrophenoxy)(phenoxy)phosphoryl)-*L*-alaninate.


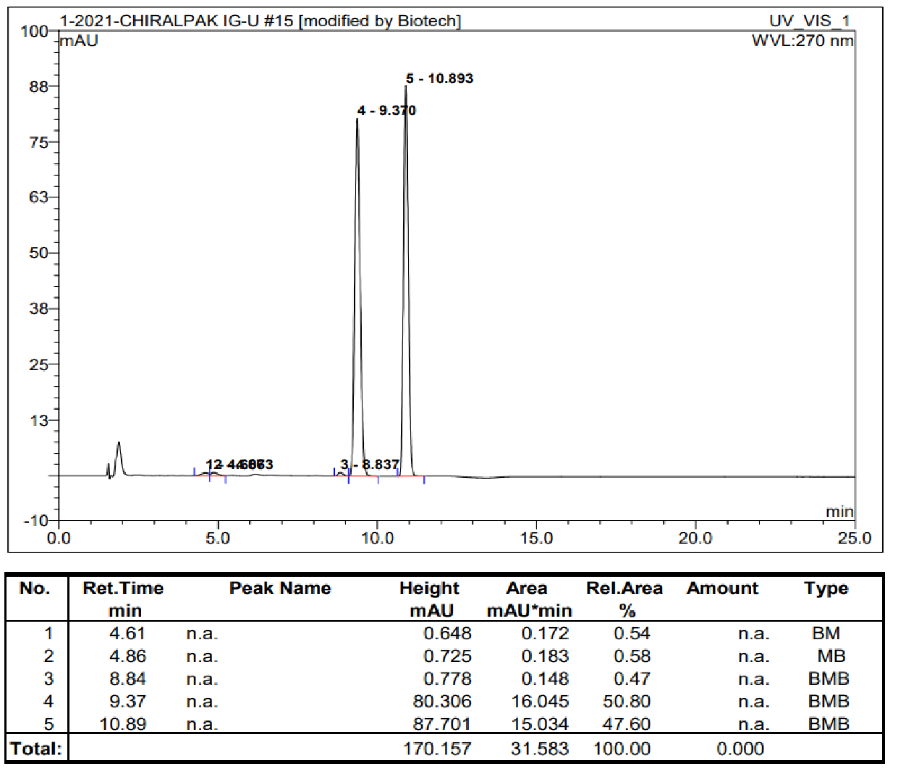


**Supplementary Figure 10.** HPLC of 2-ethylbutyl ((4-nitrophenoxy)(phenoxy)phosphoryl)-*L*-alaninate.

**Supplementary Figure 11.** ^1^H NMR of *S*p-isomer isopropyl ((4-nitrophenoxy)(phenoxy) phosphoryl)-*L*-alaninate, (*S*p)-sof.

**Supplementary Figure 12.** ^13^C NMR of *S*p-isomer isopropyl ((4-nitrophenoxy)(phenoxy) phosphoryl)-*L*-alaninate, (*S*p)-sof.

**Supplementary Figure 13.** ^31^P NMR of *S*p-isomer isopropyl ((4-nitrophenoxy)(phenoxy) phosphoryl)-*L*-alaninate, (*S*p)-sof.

**Supplementary Figure 14.** ^1^H NMR of *S*p-isomer 2-ethylbutyl ((4-nitrophenoxy)(phenoxy) phosphoryl)-*L*-alaninate, (*S*p)-rem.

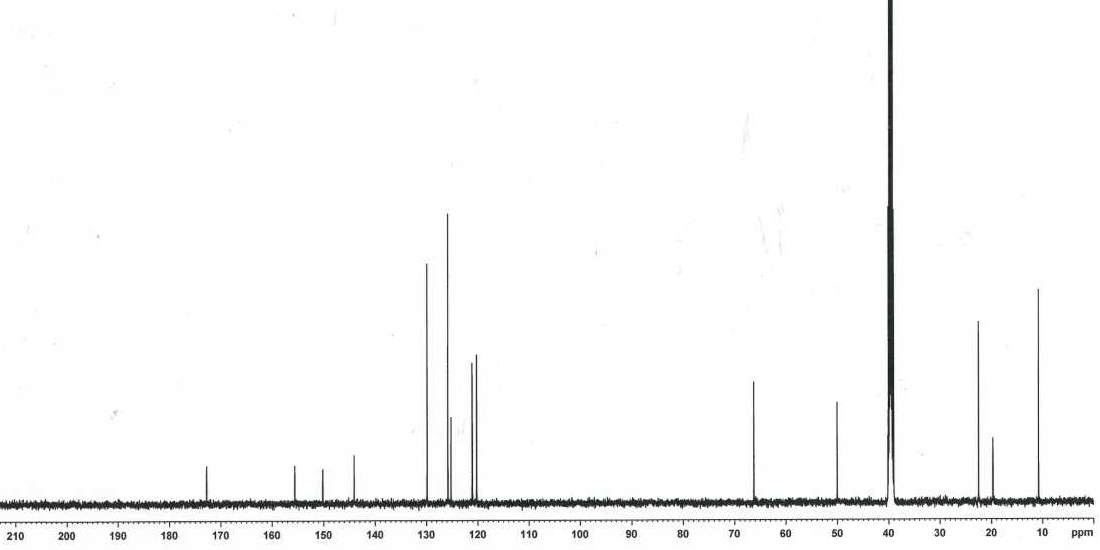


**Supplementary Figure 15.** ^13^C NMR of *S*p-isomer 2-ethylbutyl ((4-nitrophenoxy)(phenoxy) phosphoryl)-*L*-alaninate, (*S*p)-rem.

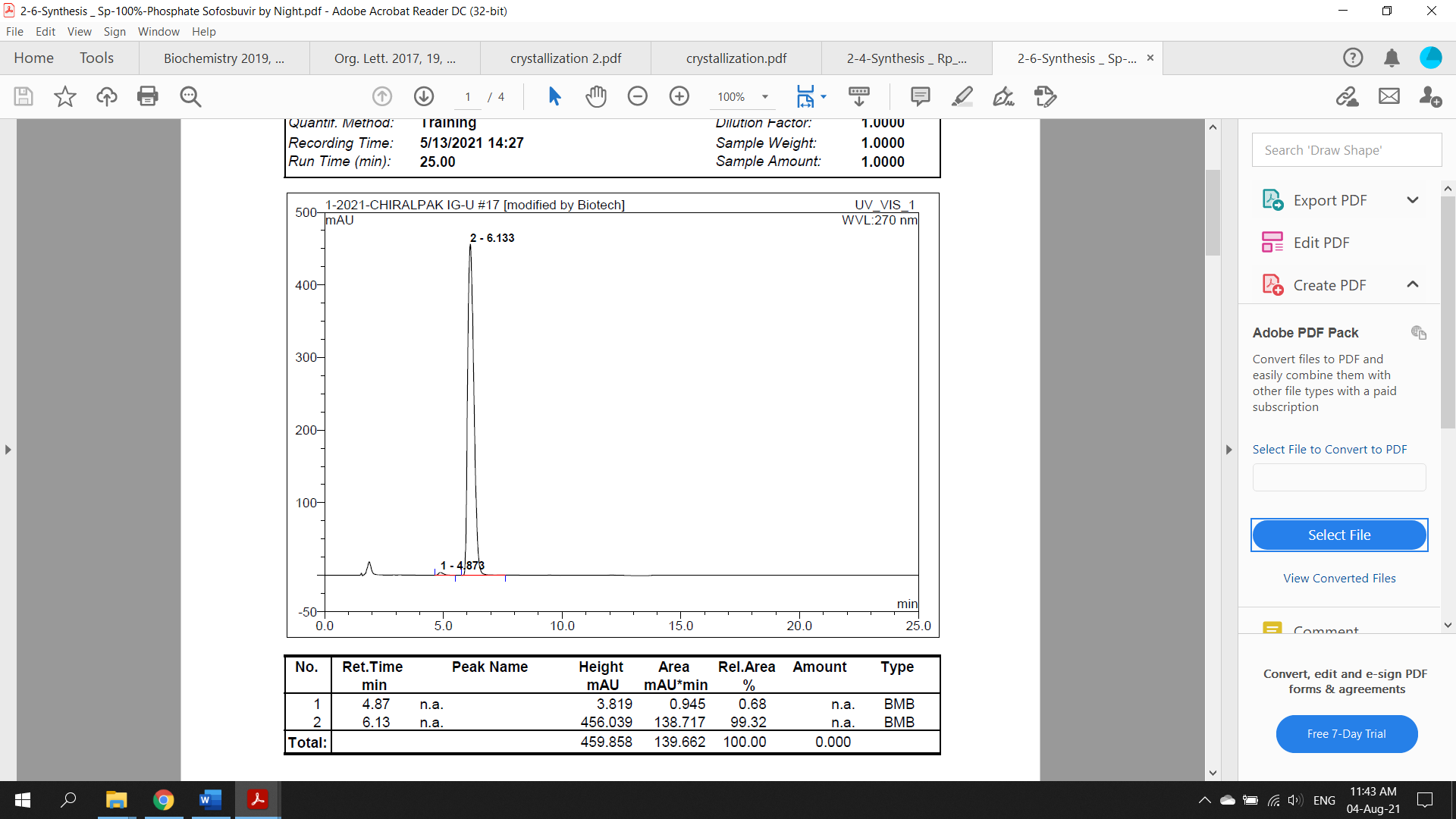
**Supplementary Figure 16.** ^31^P NMR of *S*p-isomer 2-ethylbutyl ((4-nitrophenoxy)(phenoxy) phosphoryl)-*L*-alaninate, (*S*p)-rem.

**Supplementary Figure 17.** HPLC of *S*p-isomer isopropyl ((4-nitrophenoxy)(phenoxy) phosphoryl)-*L*-alaninate, (*S*p)-sof.


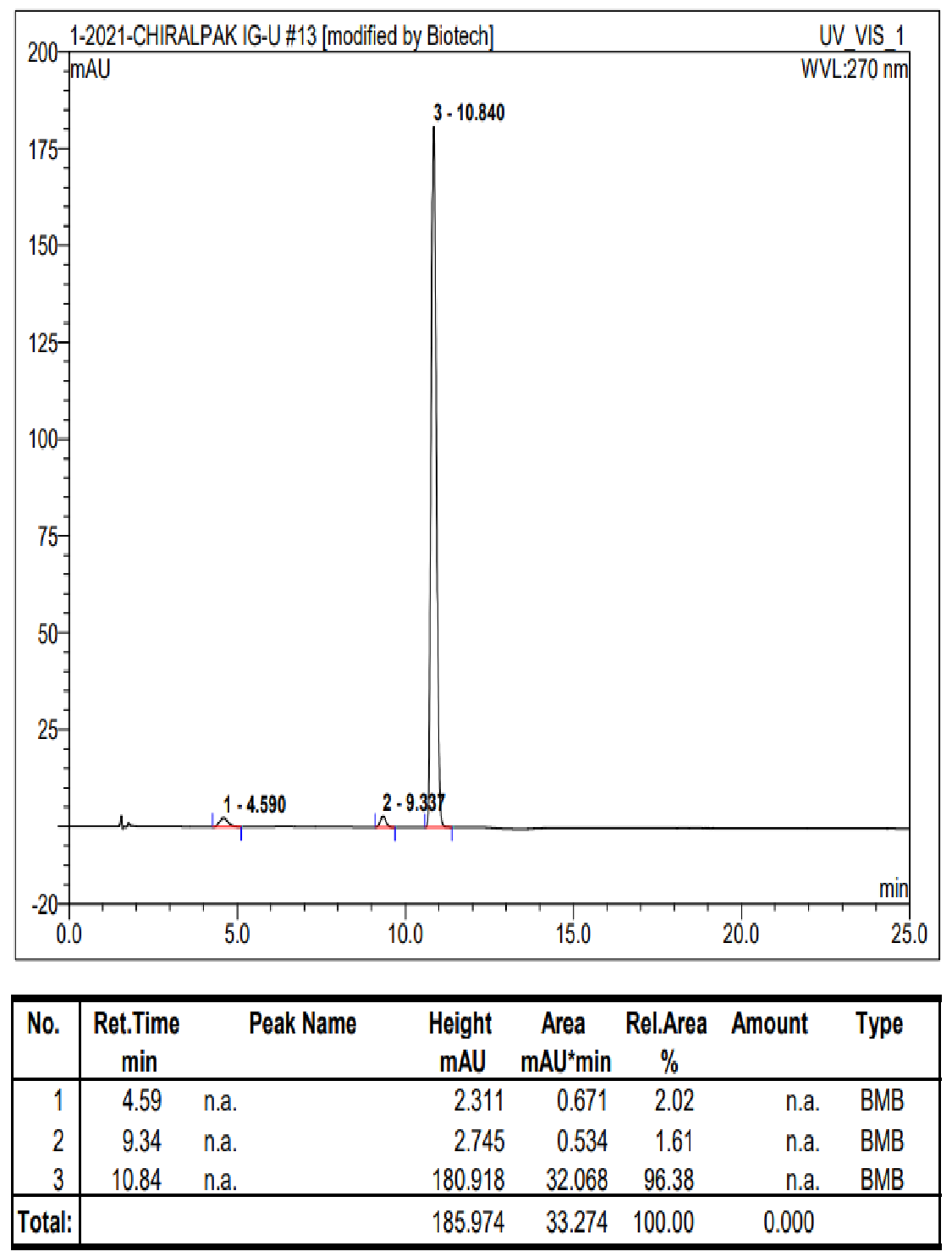


**Supplementary Figure 18.** HPLC of *S*p-isomer 2-ethylbutyl ((4-nitrophenoxy)(phenoxy) phosphoryl)-*L*-alaninate, (*S*p)-rem.

**2.3b**

**Supplementary Figure 19.** ^1^H NMR of *R*p-isomer isopropyl ((4-nitrophenoxy)(phenoxy) phosphoryl)-*L*-alaninate [(*R*p)-sof] from enzymatic approach.

**2.3b**

**Supplementary Figure 20.** ^31^P NMR of *R*p-isomer isopropyl ((4-nitrophenoxy)(phenoxy) phosphoryl)-*L*-alaninate [(*R*p)-sof] from enzymatic approach.

**2.6b**

**Supplementary Figure 21.** ^1^H NMR of *R*p-isomer 2-ethylbutyl ((4-nitrophenoxy)(phenoxy) phosphoryl)-*L*-alaninate [(*R*p)-rem] from enzymatic approach.

**2.6b**

**Supplementary Figure 22.** ^31^P NMR of *R*p-isomer 2-ethylbutyl ((4-nitrophenoxy)(phenoxy) phosphoryl)-*L*-alaninate [(*R*p)-rem] from enzymatic approach.


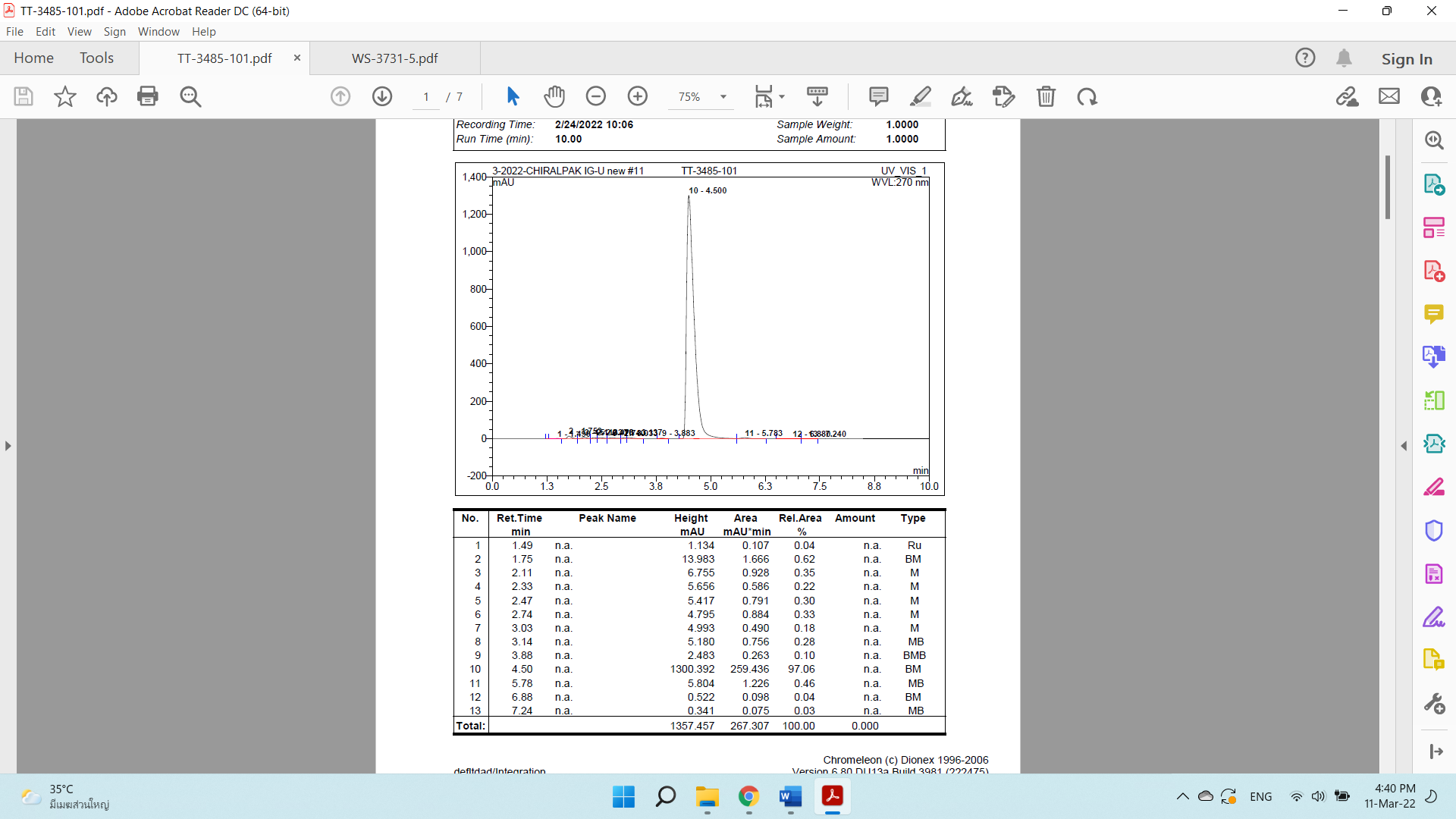


**Supplementary Figure 23.** HPLC of *R*p-isomer isopropyl ((4-nitrophenoxy)(phenoxy) phosphoryl)-*L*-alaninate [(*R*p)-sof] from enzymatic approach.


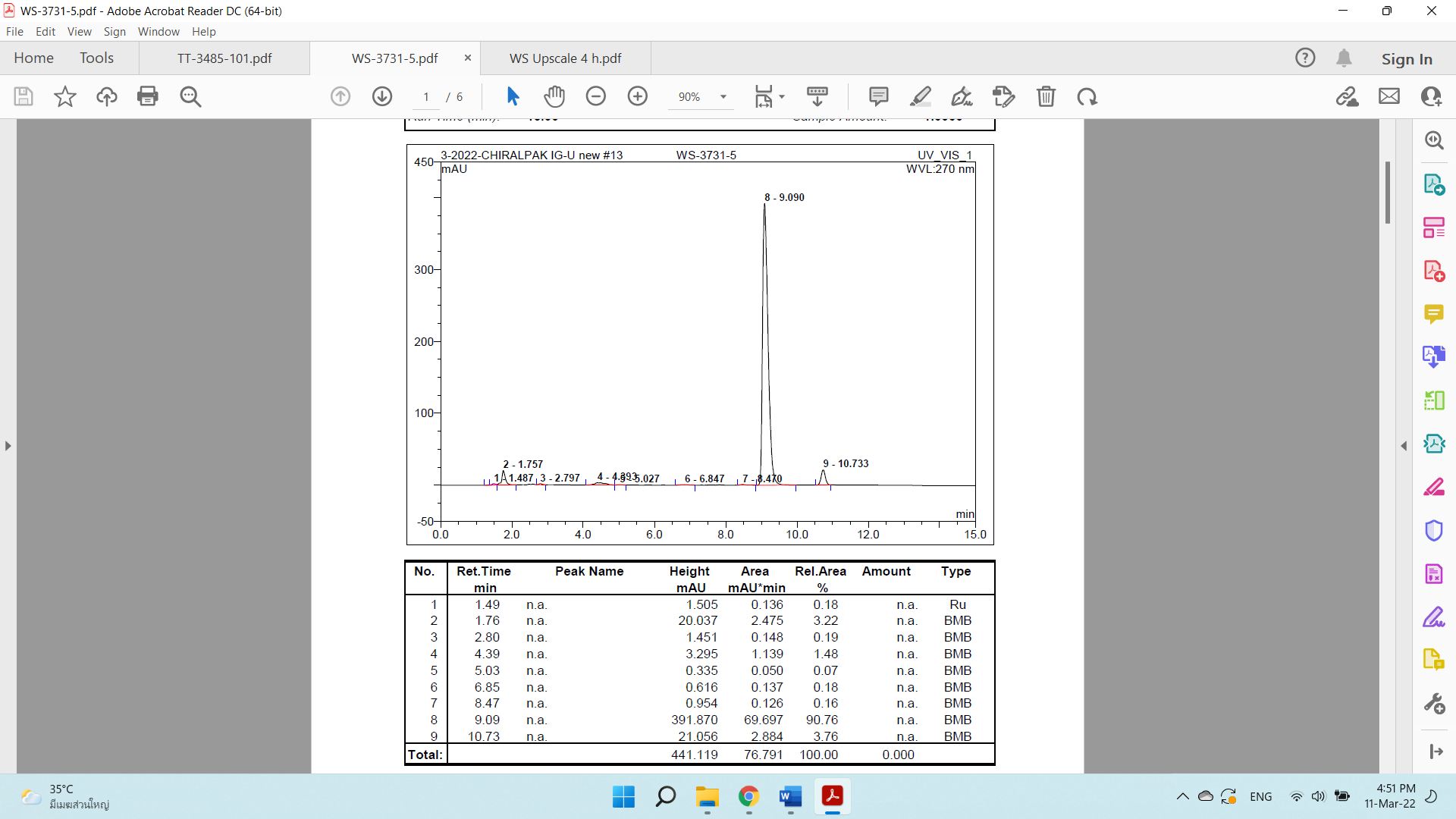


**Supplementary Figure 24.** HPLC of *R*p-isomer 2-ethylbutyl ((4-nitrophenoxy)(phenoxy) phosphoryl)-*L*-alaninate [(*R*p)-rem] from enzymatic approach.


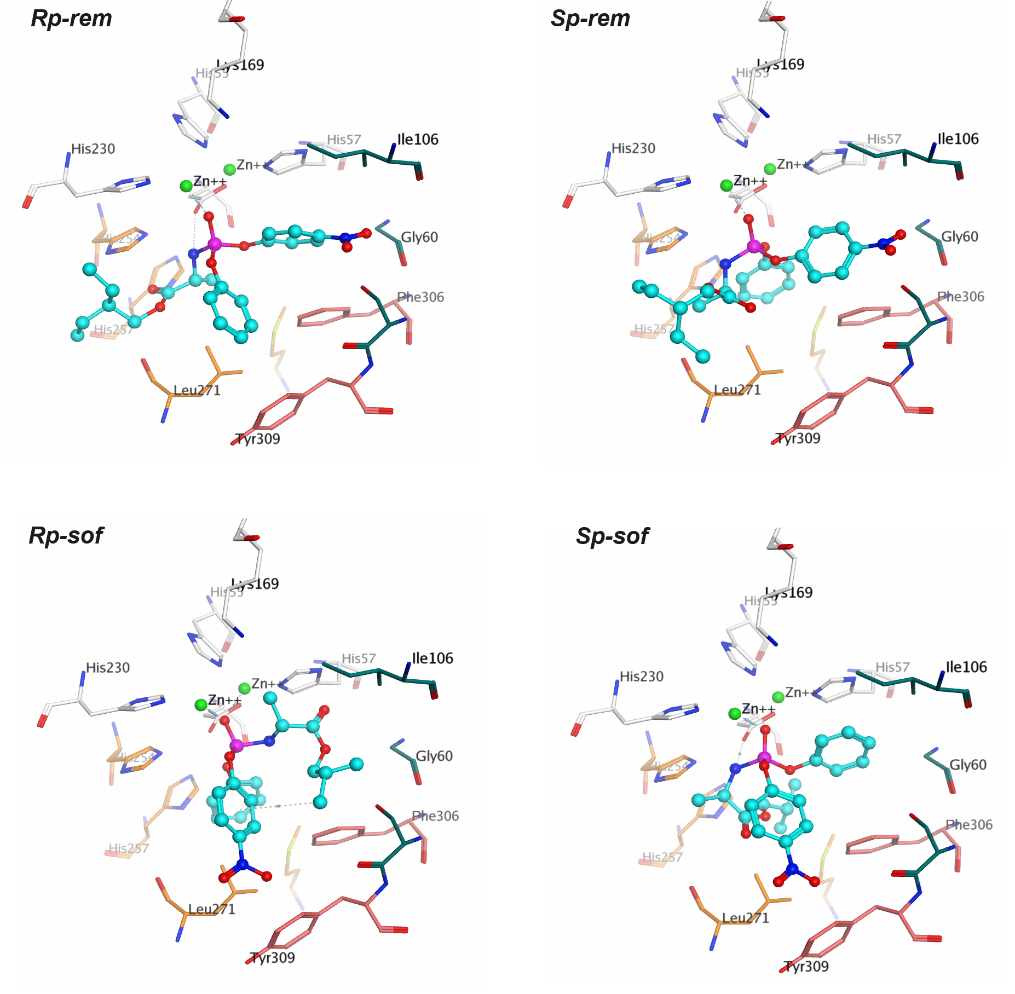


**Supplementary Figure 25.** Docking poses of *R*p- and *S*p- remdesivir and sofosbuvir precursors in WT-PTE. Pictures were generated using MOE software.


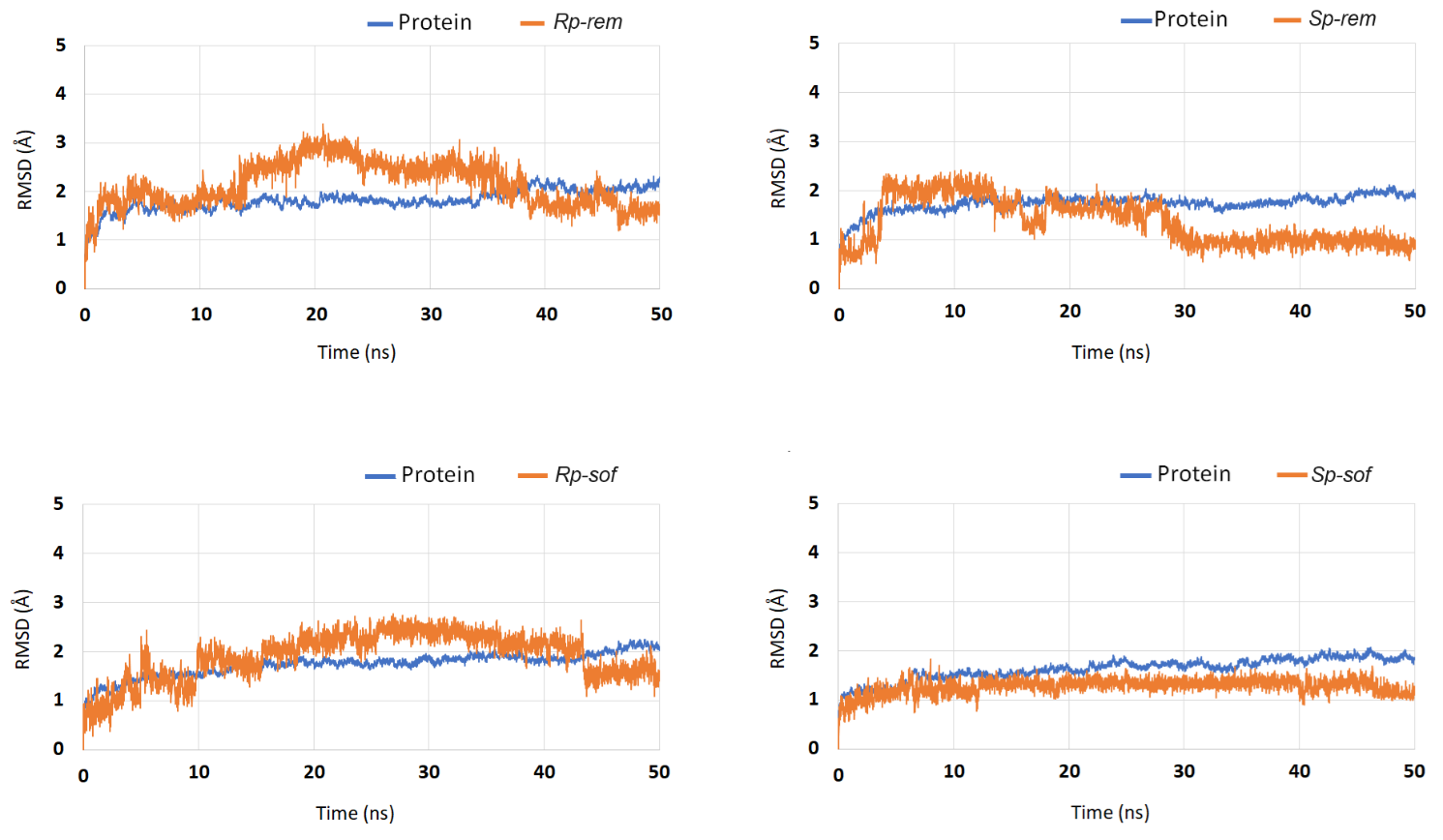


**Supplementary Figure 26.** RMSD analysis of the MD simulation trajectory. RMSD plot obtained for WT-PTE with *R*p- or *S*p- remdesivir and sofosbuvir precursors. The 50 ns simulation time showed the formation of stable complex without any significant conformational changes in the protein and ligand structures.

## Supplementary Tables

**Supplementary Table 1.** Primers used for site-directed mutagenesis.

| **No** | **Site of mutation** | **Pairs of primers** | |
| --- | --- | --- | --- |
|  |  | **Forward primer** | **Reverse primer** |
| **1** | I106A | TCGACTTTCGATGCCGGTCGCGACGTC | GACGTCGCGACCGGCATCGAAAGTCGA |
| **2** | I106V | TCGACTTTCGATGTCGGTCGCGACGTC | GACGTCGCGACCGACATCGAAAGTCGA |
| **3** | W131M | TGGCGTGCGAACATGTGTCGATGTGTC | GACACATCGACACATGTTCGCACGCCA |
| **4** | D233P | GGTCACAGCGATCCTACTGACGATTTG | CAAATCGTCAGTAGGATCGCTGTGACC |
| **5** | D233L | GGTCACAGCGATTTAACTGACGATTTG | CAAATCGTCAGTTAAATCGCTGTGACC |
| **6** | D233I | GGTCACAGCGATATTACTGACGATTTG | CAAATCGTCAGTAATATCGCTGTGACC |
| **7** | D233F | GGTCACAGCGATTTTACTGACGATTTG | CAAATCGTCAGTAAAATCGCTGTGACC |
| **8** | L271E | AGTGCATCAGCCGAACTGGGCATCCGT | ACGGATGCCCAGTTCGGCTGATGCACT |
| **9** | L271N | AGTGCATCAGCCAATCTGGGCATCCGT | ACGGATGCCCAGATTGGCTGATGCACT |
| **10** | L271F | AGTGCATCAGCCTTCCTGGGCATCCGT | ACGGATGCCCAGGAAGGCTGATGCACT |
| **11** | L303A | TCGAATGACTGGGCGTTCGGGTTTTCG | CGAAAACCCGAACGCCCAGTCATTCGA |
| **12** | L303F | TCGAATGACTGGTTTTTCGGGTTTTCG | CGAAAACCCGAAAAACCAGTCATTCGA |
| **13** | H257Y | ATCCCGTACAGTGCGATTGGTC | GACCAATCGCACTGTACGGGAT |
| **14** | S308A | TTCGGGTTTTCGGCCTATGTCACCAAC | GTTGGTGACATAGGCCGAAAACCCGAA |
| **15** | S308Y | TTCGGGTTTTCGTACTATGTCACCAAC | GTTGGTGACATAGTACGAAAACCCGAA |
| **16** | G60A | GAGCACATCTGCGCCAGCTCGGCAGGA | TCCTGCCGAGCTGGCGCAGATGTGCTC |
| **17** | F-*Nde*I and R-*Hind*III | TTTTCATATGGGCGATCGGATCAATACCGTGC | TTTTAAGCTTTCATGACGCCCGCAAGGTCGGT |

The underlined sequences correspond to the *Nde*I and *Hind*III restriction sites.

**Supplementary Table 2.** Enzyme amount for determination of enzyme activity and kinetic enzyme.

| **Recombinant enzyme** | **Protein concentration for**  **enzyme activity (µM)** | | **Protein concentration for**  **kinetic enzyme (µM)** | | | |
| --- | --- | --- | --- | --- | --- | --- |
|  | ***R*p/*S*p-sofosbuvir precursor** | ***R*p/*S*p-remdesivir precursor** | **sofosbuvir precursor** | | **remdesivir precursor** | |
|  |  |  | ***S*p-isomer** | ***R*p-isomer** | ***S*p-isomer** | ***R*p-isomer** |
| WT | 0.4000 | 3.9996 | 0.4000 | 0.4000 | 3.9996 | 3.9996 |
| G60A | 0.8445 | 0.8445 | 2.1113 | 0.8445 | 2.1113 | 0.8445 |
| I106A | 0.1039 | 0.8314 | 0.2079 | 0.0831 | 0.8314 | 0.4157 |
| W131M | 0.0036 | 0.0089 | 0.1783 | 0.0018 | 0.8917 | 0.0036 |
| G60A/I106A | 0.3331 | 1.6654 | 3.3308 | 0.3331 | 3.3308 | 0.3331 |
| G60A/W131M | 0.0125 | 0.1246 | 0.2492 | 0.0125 | 2.4916 | 0.0623 |
| I106A/W131M | 0.0362 | 0.0905 | 1.8097 | 0.0362 | 3.6193 | 0.0181 |

**Supplementary Table 3.**  Estimation of binding free energies in kcal/mol using Generalized Born accessibility algorithms (MMGB-SA). The intermolecular binding free energies were estimated using MMGB-SA by processing 1000 frames of the last 2ns of the 50 ns simulation.

| **PTE** | **∆G_total_** | | | |
| --- | --- | --- | --- | --- |
|  | **Remdesivir** | | **Sofosbuvir** | |
|  | *R*p | *S*p | *R*p | *S*p |
| WTlikePTE2 | -37.2 | -37.8 | -38.4 | -31.0 |
| In1W | -38.6 | -50.1 | -39.5 | -45.3 |
| **Small pocket** | | | | |
| G60A | -38.7 | -37.6 | -38.5 | -29.6 |
| I106A | -42.6 | -46.4 | -45.6 | -39.5 |
| I106V | -35.9 | -30.2 | -46.3 | -41.1 |
| L303A | -35.7 | -34.7 | -35.8 | -27.3 |
| L303F | -31.5 | -30.8 | -30.5 | -30.2 |
| S308A | -29.9 | -30.3 | -33.2 | -30.0 |
| S308Y | -33.1 | -31.2 | -38.4 | -34.7 |
| **Large pocket** | | | | |
| H254Y | -31.3 | -37.0 | -38.3 | -34.5 |
| L271E | -25.7 | -26.8 | -28.9 | -36.8 |
| L271N | -32.0 | -36.8 | -37.8 | -32.6 |
| L271F | -28.6 | -27.3 | -28.1 | -23.3 |
| **Leaving pocket** | | | | |
| W131M | -51.8 | -34.0 | -47.5 | -33.4 |
| D233P | -25.0 | -32.5 | -30.6 | -42.3 |
| D233L | -30.9 | -35.2 | -40.2 | -44.1 |
| D233I | -35.3 | -35.4 | -32.3 | -43.1 |
| D233F | -27.0 | -28.6 | -27.9 | -40.8 |
| G60A/I106A | -46.4 | -47.6 | -41.4 | -39.1 |
| G60A/W131M | -46.0 | -49.5 | -43.8 | -34.5 |
| I106A/W131M | -47.5 | -45.8 | -46.2 | -42.5 |
| I106A/H254Y | -35.5 | -41.5 | -41.8 | -39.1 |

Reference

D.A. Case, H.M.A., K. Belfon, I.Y. Ben-Shalom, J.T. Berryman, S.R. Brozell, D.S. Cerutti, T.E. Cheatham, Iii, G.A. Cisneros, V.W.D. Cruzeiro, T.A. Darden, N. Forouzesh, G. Giambaşu, T. Giese, M.K. Gilson, H. Gohlke, A.W. Goetz, J. Harris, S. Izadi, S.A. Izmailov, K. Kasavajhala, M.C. Kaymak, E. King, A. Kovalenko, T. Kurtzman, T.S. Lee, P. Li, C. Lin, J. Liu, T. Luchko, R. Luo, M. Machado, V. Man, M. Manathunga, K.M. Merz, Y. Miao, O. Mikhailovskii, G. Monard, H. Nguyen, K.A. O’hearn, A. Onufriev, F. Pan, S. Pantano, R. Qi, A. Rahnamoun, D.R. Roe, A. Roitberg, C. Sagui, S. Schott-Verdugo, A. Shajan, J. Shen, C.L. Simmerling, N.R. Skrynnikov, J. Smith, J. Swails, R.C. Walker, J. Wang, J. Wang, H. Wei, X. Wu, Y. Wu, Y. Xiong, Y. Xue, D.M. York, S. Zhao, Q. Zhu, and P.A. Kollman (2022). "Amber 2022". (University of California, San Francisco.).

Maier, J.A., Martinez, C., Kasavajhala, K., Wickstrom, L., Hauser, K.E., and Simmerling, C. (2015). ff14SB: Improving the Accuracy of Protein Side Chain and Backbone Parameters from ff99SB. *Journal of Chemical Theory and Computation* 11**,** 3696-3713.

Wu, X., and Brooks, B.R. (2011). Force-momentum-based self-guided Langevin dynamics: A rapid sampling method that approaches the canonical ensemble. *The Journal of Chemical Physics* 135**,** 204101.
